# Characterization of H7 Influenza A Virus in Wild and Domestic Birds in Korea

**DOI:** 10.1371/journal.pone.0091887

**Published:** 2014-04-28

**Authors:** Hyun-Mi Kang, Ha-Young Park, Kyu-Jun Lee, Jun-Gu Choi, Eun-Kyoung Lee, Byung-Min Song, Hee-Soo Lee, Youn-Jeong Lee

**Affiliations:** Avian Disease Division, Animal and Plant Quarantine Agency, Anyangsi, Gyeonggido, Republic of Korea; University of Maryland, United States of America

## Abstract

During surveillance programs in Korea between January 2006 and March 2011, 31 H7 avian influenza viruses were isolated from wild birds and domestic ducks and genetically characterized using large-scale sequence data. All Korean H7 viruses belonged to the Eurasian lineage, which showed substantial genetic diversity, in particular in the wild birds. The Korean H7 viruses from poultry were closely related to those of wild birds. Interestingly, two viruses originating in domestic ducks in our study had the same gene constellations in all segment genes as viruses originating in wild birds. The Korean H7 isolates contained avian-type receptors (Q226 and G228), no NA stalk deletion (positions 69–73), no C-terminal deletion (positions 218–230) in NS1, and no substitutions in PB2-627, PB1-368, and M2-31, compared with H7N9 viruses. In pathogenicity experiments, none of the Korean H7 isolates tested induced clinical signs in domestic ducks or mice. Furthermore, while they replicated poorly, with low titers (10 ^0.7–1.3^EID_50_/50 µl) in domestic ducks, all five viruses replicated well (up to 7–10 dpi, 10 ^0.7–4.3^EID_50_/50 µl) in the lungs of mice, without prior adaptation. Our results suggest that domestic Korean viruses were transferred directly from wild birds through at least two independent introductions. Our data did not indicate that wild birds carried poultry viruses between Korea and China, but rather, that wild-type H7 viruses were introduced several times into different poultry populations in eastern Asia.

## Introduction

H7 influenza A virus (IAV) circulates continuously in wild birds worldwide [13], [27], [28], [40]. In domestic birds, H7 IAVs may become highly pathogenic following introduction into poultry from wild birds and cause outbreaks of highly pathogenic avian influenza (HPAI) [9]. Since 2002, H7 IAVs have sporadically infected humans, causing more than 100 cases of human infection. Most of the cases showed mild clinical signs with conjunctivitis, with the exception of one fatal case in the Netherlands [3], [9], [18], [29].

In the spring of 2013, H7N9 IAVs causing human infections were first identified in China, and by 12 August 2013, this virus had infected 135 people in China and Taiwan, and killed 44 [43]. It is believed that the H7N9 virus resulted from a reassortment of at least three avian influenza strains [30], [35], [39]. Unlike the H5N1 viruses, which are well characterized in terms of their genetic and pathogenic characteristics, limited information is available for H7-subtype viruses in Asia. Since the emergence of H7N9 viruses, many articles tracing their sources have been published in China [30], [35], [39]. While the source of human infection by H7N9 virus cannot be verified, available evidence suggests that it is likely to have been introduced via poultry or contaminated environments in live bird markets [4].

In South Korea, active surveillance has been applied to wild birds and domestic birds since the first H5N1 HPAI outbreak in 2003–2004. A variety of IAVs, including H5 and H7 low pathogenic avian influenza (LPAI) viruses, have been isolated in wild birds [26], with the prevalence of H5 and H7 viruses relatively high; 10.8% and 5.8% respectively [23]. H7 viruses have also been detected in domestic birds. From 2009–2011, nine H7 viruses were isolated from domestic ducks, and these nine were closely related to viruses isolated from wild birds. Since then, however, H7 viruses have not been reported in domestic birds in Korea [25].

In this study, we characterized H7 IAVs (31 isolates) from wild birds and two domestic ducks in Korea between January 2006 and March 2011 through surveillance programs. We attempted to elucidate relationships between H7 viruses from wild birds and poultry from Korea and elsewhere in Asia and Europe by comparing their genetic characteristics. Focusing on the Eurasian-origin viruses, we conducted large-scale genetic analyses to elucidate the origin of the Korean H7 viruses in domestic ducks. In addition, we evaluated the pathogenic potential of these IAVs using animal experiments performed in domestic ducks and in mice.

## Materials and Methods

### Virus isolation

Through active surveillance programs in South Korea between January 2006 and March 2011, 31 H7 IAVs were isolated from duck farms (oropharyngeal [OP] and cloacal swab samples) and more than 30 wild bird habitats (fecal samples) located mainly along the western and southern plain regions of South Korea where migratory birds aggregate. Although it cannot be established with certainty that the birds were migratory rather than resident, the fecal samples were collected from habitats that serve as overwintering locales for migratory wild birds arriving from northern Asia, including Russia and Mongolia, during September to the following March [23].

This study did not involve endangered or protected species; only fecal samples from migratory birds were collected. Each sample was suspended in an antibiotic phosphate buffered saline (PBS) solution and centrifuged at 3,500 rpm (HERAEUS MULTIFUGE X3R centrifuge, Thermo SCIENTIFIC) for 5 min. The supernatants were inoculated into the allantoic cavities of 9- to 11-day-old embryonated hen eggs and incubated at 37°C for 4–5 days. After incubating the eggs, the allantoic fluid was harvested and centrifuged for purification. Virus presence was determined using a hemagglutination assay, and the detected viruses were subtyped by reverse transcription (RT)-PCR using influenza-specific primers [33] and Maxime RT-PCR PreMix (iNtRON, Seongnam, South Korea).

### Identification of bird species

As fecal samples, rather than captured bird samples, were used for AI surveillance, AI prevalence or subtypes could not be correlated with bird species prior to 2007. However, after 2007, it was possible to identify host species, via the barcoding system, using mitochondrial DNA recovered from fecal samples [16], [32]. Mitochondrial DNA was extracted from bird fecal samples using the DNA Stool Mini Kit (Qiagen, Valencia, CA). The mitochondrial cytochrome c oxidase I gene was amplified by universal and modified primers [32] using the AccuPower™ PCR PreMix (Bioneer, Daejeon, South Korea). The PCR products were sequenced and identified using information from the Barcode of Life Data Systems website (http://www.barcodinglife.org).

### Molecular analyses

Viral RNA was extracted from virus-containing allantoic fluid of embryonated eggs using the Viral Gene-spin viral DNA/RNA extraction kit (iNtRON, Seongnam, South Korea). RT-PCR was performed using the QIAGEN OneStep RT-PCR Kit (QIAGEN, Valencia, CA) with segment-specific primers [19] and the PCR products were purified using the QIAquick gel extraction kit (QIAGEN, Valencia, CA). Sequencing reactions were carried out using an ABI 3730 XL sequencer (Applied Biosystems, Foster City, CA). Sequences were aligned and edited using the Vector NTI Advance program (Invitrogen, Carlsbad, CA). Molecular characteristics were deduced after translation into the amino acid sequence using the MEGA 5.2 program [37]. For phylogenetic analysis, the nucleotide sequences of all eight genome segments were used. Some of these viral genes have been previously described [25], [26]; the remaining genes were analyzed and the sequences were deposited in GenBank (KC609764–KC609769, KC609771–KC609838, KC609840–KC609869, KC609872–KC609979). The sequences of recent H7N9 viruses used for genetic comparison of the hemagglutinin (HA) gene (subtype H7) and internal genes (subtypes H7 and H9) were focused on the Eurasian lineage. They were downloaded from the Influenza Sequence Database (ISD) of GenBank, the Influenza Database, and the Global Initiative on Sharing Avian Influenza Data (GISAID) Database. Several virus sequences of the American lineage were also downloaded to use as an outgroup in phylogenetic trees. For the viruses of the American lineage, N2 gene sequences were downloaded. Phylogenetic trees were constructed using the maximum likelihood method with general time-reversible model, invariant sites, and 4 gamma-distributed heterogeneous substitution rates (GTR+ I + Γ4 model) and 100 bootstrap replications in PhyML 3.0 [11]. Statistical support for the phylogenies was assessed by the approximate likelihood test using a Shimodaira-Hasegawa-like procedure in PhyML 3.0. The topology of the trees was visualized in FigTree 1.4.

### Ethics statement

This study was carried out in strict accordance with the recommendations in the Guidelines of the Institutional Animal Care and Use Committee of the Animal and Plant Quarantine Agency (IACUC, QIA) in Korea (approval number: 2011–058). All mice autopsies were performed under anesthesia with Zoletil (Virbac S.A., France), and all efforts were made to minimize suffering. This study did not affect endangered or protected species because only fecal samples of migratory birds were collected.

### Animal experiment in domestic ducks and mice

To assess pathogenicity in domestic ducks and mice, 2-week-old domestic ducks (Pekin ducks, Korea) and 6-week-old BALB/c mice (Orient Bio, Korea) were inoculated intranasally with each virus. The following host-virus combinations were included in the experiments: A/duck/Korea/BC10/2007 (H7N3), A/duck/Korea/GJ56/2007 (H7N8), A/wild duck/Korea/MHC35-41/2011 (H7N9), A/wild duck/Korea/CSM27-12/2009 (H7N6), and A/common teal/Korea/MHC5-8/2009 (H7N7). Domestic ducks and mice were observed for clinical signs during the experiments. To determine virus shedding, OP and cloacal swab samples in domestic ducks were collected from each group of nine domestic ducks on days 1, 3, 5, 7, and 10 post-inoculation. To determine virus replication in mice, lung tissue samples were collected on days 1, 3, 5, 7, and 10 post-inoculation, from two euthanized mice per group. To elucidate patterns of virus replication in major tissues of the infected host, two domestic ducks per group were euthanized and tissue samples (brain, trachea, lung, cecal tonsil, kidney, and spleen) were collected at 3 and 7 days post-inoculation (dpi). For virus isolation, each swab sample was suspended in 1 ml of sterilized PBS containing 1% gentamicin and each tissue sample was homogenized in PBS with antibiotics to a final concentration of 10% wt/vol. Samples were then centrifuged at 3,500 rpm for 5 min and each 0.1 ml supernatant was inoculated into the allantoic cavities of 9–11-day-old embryonated hen eggs, which were then incubated at 37°C for 4–5 days. Allantoic fluid from the incubated eggs was harvested and centrifuged for purification. Virus presence was determined by hemagglutination assay.

### Antigenic analyses

Antigenic analyses were performed by a hemagglutinin inhibition (HI) test using chicken antisera generated against the tested viruses, as described previously [45]. To generate the antisera, 4-week-old specific pathogen free (SPF) chickens were injected with 0.5 ml of oil emulsion-inactivated virus, and sera were collected 2 weeks after infection. The HI test used four hemagglutination units (HAU) of antigens and 1% chicken erythrocytes, and the *r*-value was subsequently calculated as described previously [1].

## Results

### Isolation of the Korean H7 viruses

All viruses in this study were isolated by the QIA of South Korea through active surveillance programs between January 2006 and March 2011. Within this collection, 22,277 samples were collected and 216 influenza viruses were recovered, representing a prevalence of 0.97%. Influenza viruses subtypes H1–H13 and N1–N9 were isolated from samples. Of 216 viruses, H6 (19.0%, n = 41) and H4 (18.5%, n = 40) were the most abundantly detected HA subtypes, followed by H7 (14.4%, n = 31), H9 (10.2%, n = 22), H1 (9.3%, n = 20), H5 (8.3%, n = 18), H3 (7.9%, n = 17), H11 (4.2%, n = 9), H10 (3.7%, n = 8), H2 (2.3%, n = 5), H12 (0.9%, n = 2), and H8, H13, and mixed virus (0.5%, n = 1). Thirty-one H7 viruses were isolated from two domestic ducks (living on farms) without clinical signs and 29 fecal samples from various wild birds, including mallard (*Anas platyrhynchos*) (27.6%), common teal (*Anas crecca*) (3.4%), Northern shoveler (*Anas clypeata*) (3.4%), other wild duck (48.3%), bean goose (*Anser fabalis*) (3.4%), magpie (*Pica pica serica*) (3.4%), and unidentified species (10.3%). The NA genes had various subtypes: N7 (39.7%), N9 (35.5%), N3 (12.9%), N8 (6.5%), N2 (3.2%), and N6 (3.2%) (Table 1).

**Table 1:** Comparison of the signature amino acids in the proteins of Korean H7 isolates in wild birds and poultry with H7N9 in China.

	Strain	Date of	Host species*	Sample	Location	Virus	HA		Cleavage site	NA	NA deletion	M2	NS deletion	PB2	PB1	PA			Reference
		isolation		type		subtype	217/226	219/228	315-321 (333/339)	294	69-73/N9	31	218-230	627	368	100	356	409	
	A/bean goose/Korea/SH20-17/2008	Dec-08	bean goose	fecal	Gyeonggido	H7N3	Q	G	PEIPKRR	R	No	S	No	E	I	V	K	S	In this study
	A/common teal/Korea/MHC5-8/2009	Oct-09	common teal	fecal	Chungbuk	H7N7	Q	G	PEIPKGR	R	No	S	No	E	I	V	K	S	In this study
	A/magpie/Korea/YJD174/2007	Dec-07	magpie	fecal	Incheon	H7N7	Q	G	PEIPKGR	R	No	S	No	E	I	V	K	S	In this study*
	A/mallard/Korea/GG1/2007	Nov-07	mallard	fecal	Jeonbuk	H7N7	Q	G	PETPKGR	R	No	S	No	E	I	V	K	S	In this study
	A/mallard/Korea/GG2/2007	Nov-07	mallard	fecal	Jeonbuk	H7N7	Q	G	PETPKGR	R	No	S	No	E	I	V	K	S	In this study*
	A/mallard/Korea/GG3/2007	Nov-07	mallard	fecal	Jeonbuk	H7N7	Q	G	PETPKGR	R	No	S	No	E	I	V	K	S	In this study*
	A/mallard/Korea/GH170/2007	Dec-07	mallard	fecal	Incheon	H7N7	Q	G	PEIPKGR	R	No	S	No	E	I	V	K	S	In this study*
	A/mallard/Korea/GH171/2007	Dec-07	mallard	fecal	Incheon	H7N7	Q	G	PEIPKGR	R	No	S	No	E	I	V	K	S	In this study*
	A/mallard/Korea/GJ62/2007	Nov-07	mallard	fecal	Gwangju	H7N2	Q	G	PEIPKGR	R	No	S	No	E	I	V	K	S	In this study
	A/mallard/Korea/GJ63/2007	Nov-07	mallard	fecal	Gwangju	H7N8	Q	G	PEIPKGR	R	No	S	No	E	I	V	K	S	In this study
	A/mallard/Korea/NHG187/2008	Jan-08	mallard	fecal	Gyeongnam	H7N7	Q	G	PEIPKGR	R	No	S	No	E	I	V	K	S	In this study
	A/Northern shoveler/SD175/2008	Jan-09	Northern shoveler	fecal	Seoul	H7N3	Q	G	PEIPKGR	R	No	S	No	E	I	V	K	S	In this study
	A/wild bird feces/Korea/HDR22/2006	Jan-06	wild bird	fecal	Jeju	H7N7	Q	G	PEIPKGR	R	No	S	No	E	I	V	K	S	In this study*
	A/wild bird feces/Korea/HDR23/2006	Jan-06	wild bird	fecal	Jeju	H7N7	Q	G	PEIPKGR	R	No	S	No	E	I	V	K	S	In this study
wild birds	A/wild bird feces/Korea/SH21/2006	Jan-06	wild bird	fecal	Gyeonggido	H7N3	Q	G	PEIPKGR	R	No	S	No	E	I	V	K	S	In this study
in Korea	A/wild duck/Korea/CSM27-12/2009	Feb-09	wild duck	fecal	Chungnam	H7N6	Q	G	PEIPKRR	R	No	S	No	E	I	V	K	S	In this study
	A/wild duck/Korea/CSM42-1/2011	Mar-11	wild duck	fecal	Chungnam	H7N9	Q	G	PELPKGR	R	No	S	No	E	I	V	K	S	In this study
	A/wild duck/Korea/CSM42-34/2011	Mar-11	wild duck	fecal	Chungnam	H7N9	Q	G	PELPKGR	R	No	S	No	E	I	V	K	S	In this study
	A/wild duck/Korea/MHC35-25/2011	Mar-11	wild duck	fecal	Chungbuk	H7N9	Q	G	PEPPKGR	R	No	S	No	E	I	V	K	S	In this study
	A/wild duck/Korea/MHC35-41/2011	Mar-11	wild duck	fecal	Chungbuk	H7N9	Q	G	PEPPKGR	R	No	S	No	E	I	V	K	S	In this study
	A/wild duck/Korea/MHC39-13/2011	Mar-11	wild duck	fecal	Chungbuk	H7N9	Q	G	PEPPKGR	R	No	S	No	E	I	V	K	S	In this study
	A/wild duck/Korea/MHC39-26/2011	Mar-11	wild duck	fecal	Chungbuk	H7N9	Q	G	PEPPKGR	R	No	S	No	E	I	V	K	S	In this study
	A/wild duck/Korea/MHC40-28/2010	Mar-10	wild duck	fecal	Chungbuk	H7N7	Q	G	PEIPKGR	R	No	S	No	E	I	V	K	S	In this study
	A/wild duck/Korea/SH19-27/2010	Nov-10	wild duck	fecal	Gyeonggido	H7N9	Q	G	PELPKGR	R	No	S	No	E	I	V	K	S	In this study
	A/wild duck/Korea/SH19-44/2010	Nov-10	wild duck	fecal	Gyeonggido	H7N9	Q	G	PELPKGR	R	No	S	No	E	I	V	K	S	In this study
	A/wild duck/Korea/SH19-47/2010	Nov-10	wild duck	fecal	Gyeonggido	H7N9	Q	G	PELPKGR	R	No	S	No	E	I	V	K	S	In this study
	A/wild duck/Korea/SH19-50/2010	Jan-10	wild duck	fecal	Gyeonggido	H7N9	Q	G	PELPKGR	R	No	S	No	E	I	V	K	S	In this study
	A/wild duck/Korea/SH20-27/2008	Dec-08	wild duck	fecal	Gyeonggido	H7N9	Q	G	PELPKGR	R	No	S	No	E	I	V	K	S	In this study
	A/wild duck/Korea/SH41-33/2011	Mar-11	wild duck	fecal	Gyeonggido	H7N7	Q	G	PELPKGR	R	No	S	No	E	I	V	K	S	In this study
	A/duck/Korea/BC10/2007	Mar-07	domestic duck	swab	Chungnam	H7N3	Q	G	PETPKGR	R	No	S	No	E	I	V	K	S	In this study**
ducks	A/duck/Korea/GJ56/2007	Nov-07	domestic duck	swab	Gwangju	H7N8	Q	G	PEIPKGR	R	No	S	No	E	I	V	K	S	In this study**
in Korea	A/duck/Korea/A117/2010	Nov-10	domestic duck	fecal	Jeonnam	H7N6	Q	G	PEIPRGR	R	No	S	No	E	I	V	K	S	Kim et al.
	A/duck/Korea/109/2011	Jan-11	domestic duck	fecal	Chungnam	H7N7	Q	G	PEIPKGR	R	No	S	No	E	I	V	K	S	Kim et al.
	Shanghai/1/13	Feb-13	human		Shanghai	H7N9	Q	G	PEIPKGR	K	Yes	N	Yes	K	I	A	R	N	GISAID
	Shanghai/2/13	Mar-13	human		Shanghai	H7N9	L	G	PEIPKGR	R	Yes	N	Yes	K	V	A	R	N	GISAID
avian	Anhui/1/13	Mar-13	human		Anhui	H7N9	L	G	PEIPKGR	R	Yes	N	Yes	K	V	A	R	N	GISAID
origin	Hangzhou/1/13	Mar-13	human		Hangzhou	H7N9	I	G	PEIPKGR	R	Yes	N	Yes	K	V	A	R	N	GISAID
H7N9	chicken/Shanghai/S1053/13	Apr-13	chicken		Shanghai	H7N9	L	G	PEIPKGR	R	Yes	N	Yes	E	V	A	R	N	GISAID
(China)	pigeon/Shanghai/S1069/13	Apr-13	pigeon		Shanghai	H7N9	L	G	PEIPKGR	R	Yes	N	Yes	E	V	A	R	N	GISAID
	environment/Shanghai/S1088/13	Apr-13	environment		Shanghai	H7N9	L	G	PEIPKGR	R	Yes	N	Yes	E	V	A	R	N	GISAID

^*^ Since 2007, a barcoding system utilising mitochondrial DNA of bird faeces has been employed to determine host species. The DNA barcoding system can identify over 98% bird species. Wild duck indicates mallard or spot-billed duck, which might have recently split their speciation and have many hybrid.

^**^one or more genes of some Korean viruses were published in a previous report [25], [26] and other remaining genes ware analyzed and deposited in Genbank (KC609764-KC609769, KC609771-KC609838, KC609840-KC609869, KC609872-KC609979).

### Phylogenetic analyses

**Figure 1:**
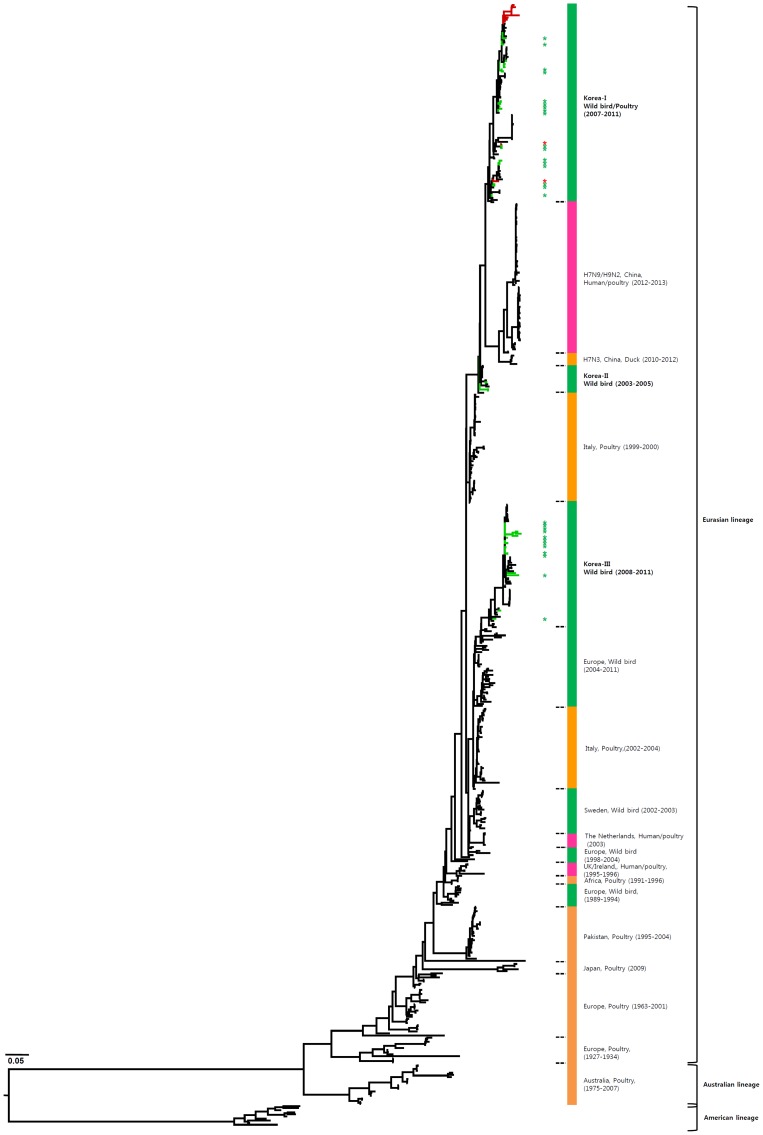
Phylogenies of H7 (*n* = 541) genes. Tip and branch colors represent host origin (wild birds in green, domestic birds in red) of all of the Korean H7 viruses, and asterisks denote the Korean H7 viruses isolated in the present study. Phylogenetic trees were constructed using the maximum likelihood method with general time-reversible model with invariant sites and 4 gamma-distributed heterogeneous substitution rates (GTR+ I + Γ4 model) and 100 bootstrap replications (H7 I = 0.285 α = 1.092; N9 I = 0.416 α = 1.452; N7 I = 0.411 α = 1.528; N3 I = 0.269 α = 0.858; N8 I = 0.371 α = 1.162; N2 I = 0.417 α = 1.590; N6 I = 0.309 α = 0.962) in PhyML 3.0 [11]. Statistical support for the phylogenies was assessed by the approximate likelihood test using a Shimodaira-Hasegawa-like procedure in PhyML 3.0. The topology of trees was visualized in FigTree 1.4. Viruses from different hosts are indicated by: wild birds, green; poultry, orange; human, pink.

The HA genes of H7 viruses can be divided into three main lineages, namely, the Eurasian, Australian, and American lineages (Figure 1 and Figure S1a), with the Eurasian lineage divided further into various sublineages. The genetic evolution of H7 viruses has been observed over time, and H7 viruses from poultry, characterized in 1927–1934 and 1963–1982, have been related to those isolated from wild birds. All of the Korean H7 viruses isolated in the present study belonged to the Eurasian lineage, which can be divided into three sublineages (Korea-I, II, and III). Korea-I was composed of viruses from wild birds (green) and poultry (red) in 2007–2011, whereas Korea-II and -III viruses were isolated only from wild birds in 2003–2005 and 2008–2011, respectively. The Korean H7 viruses in both domestic ducks and wild birds were distinct from the H7N9 viruses in China.

**Figure 2:**
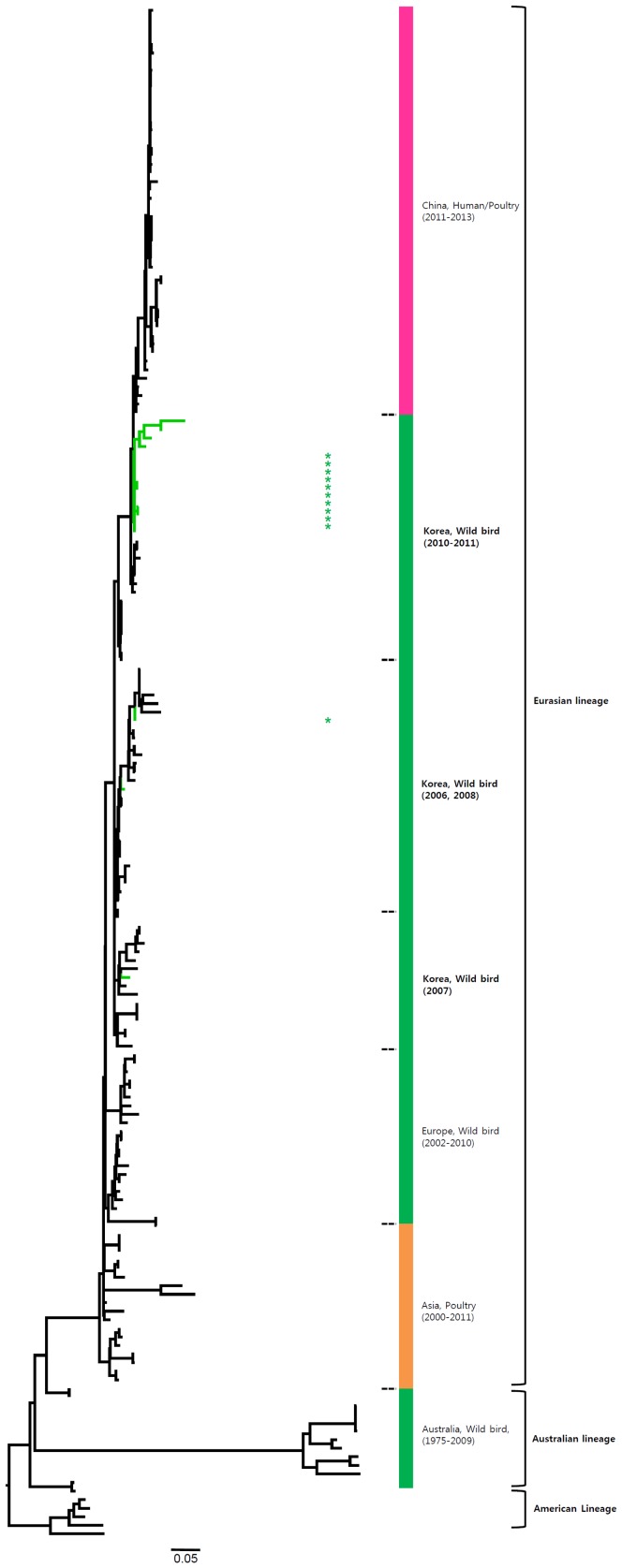
Phylogenies of N9 (*n* = 179) genes. Tip and branch colors represent host origin (wild birds in green, domestic birds in red) of all of the Korean H7 viruses, and asterisks denote the Korean H7 viruses isolated in the present study. Phylogenetic trees were constructed using the maximum likelihood method with general time-reversible model with invariant sites and 4 gamma-distributed heterogeneous substitution rates (GTR+ I + Γ4 model) and 100 bootstrap replications (H7 I = 0.285 α = 1.092; N9 I = 0.416 α = 1.452; N7 I = 0.411 α = 1.528; N3 I = 0.269 α = 0.858; N8 I = 0.371 α = 1.162; N2 I = 0.417 α = 1.590; N6 I = 0.309 α = 0.962) in PhyML 3.0 [11]. Statistical support for the phylogenies was assessed by the approximate likelihood test using a Shimodaira-Hasegawa-like procedure in PhyML 3.0. The topology of trees was visualized in FigTree 1.4. Viruses from different hosts are indicated by: wild birds, green; poultry, orange; human, pink.

**Figure 3:**
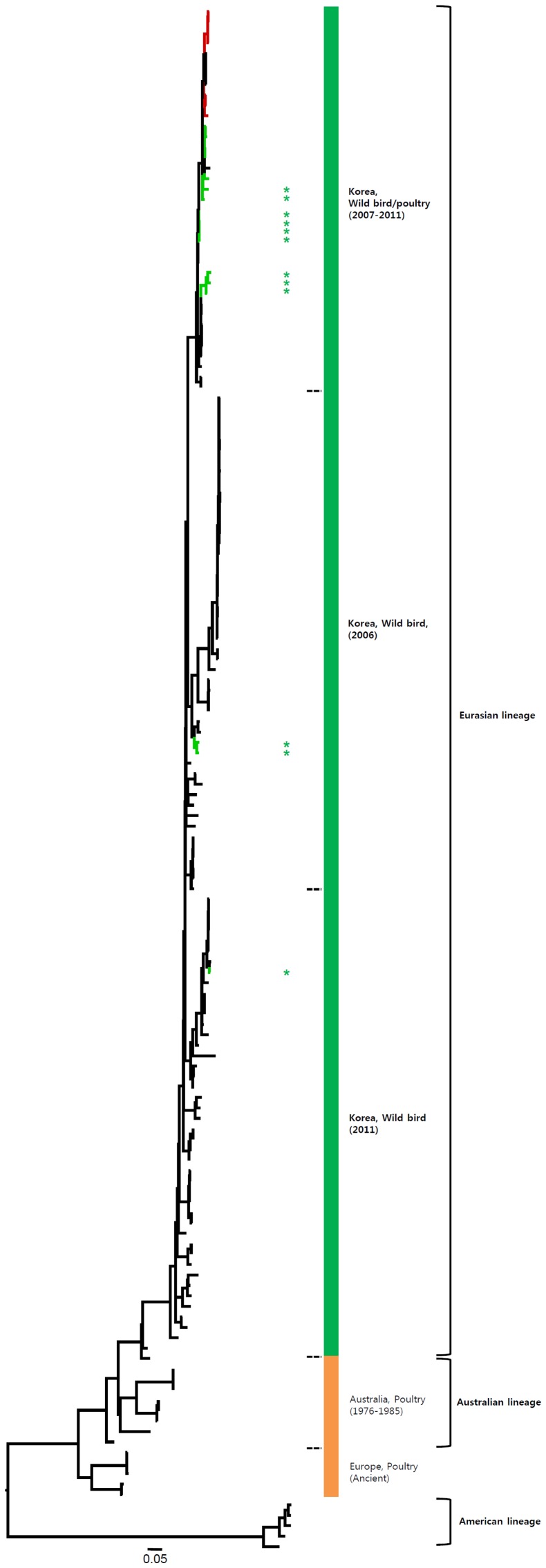
Phylogenies of N7 (*n* = 148) genes. Tip and branch colors represent host origin (wild birds in green, domestic birds in red) of all of the Korean H7 viruses, and asterisks denote the Korean H7 viruses isolated in the present study. Phylogenetic trees were constructed using the maximum likelihood method with general time-reversible model with invariant sites and 4 gamma-distributed heterogeneous substitution rates (GTR+ I + Γ4 model) and 100 bootstrap replications (H7 I = 0.285 α = 1.092; N9 I = 0.416 α = 1.452; N7 I = 0.411 α = 1.528; N3 I = 0.269 α = 0.858; N8 I = 0.371 α = 1.162; N2 I = 0.417 α = 1.590; N6 I = 0.309 α = 0.962) in PhyML 3.0 [11]. Statistical support for the phylogenies was assessed by the approximate likelihood test using a Shimodaira-Hasegawa-like procedure in PhyML 3.0. The topology of trees was visualized in FigTree 1.4. Viruses from different hosts are indicated by: wild birds, green; poultry, orange; human, pink.

**Figure 4:**
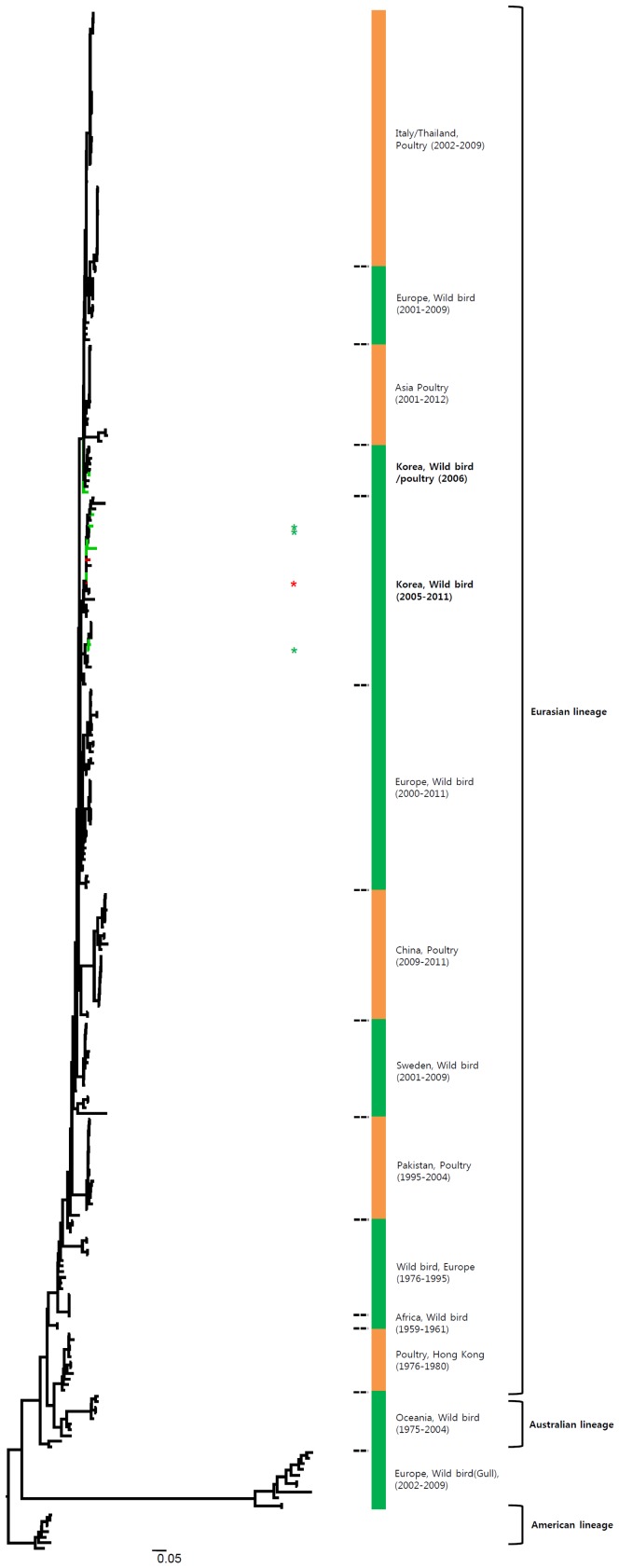
Phylogenies of N3 (*n* = 273) genes. Tip and branch colors represent host origin (wild birds in green, domestic birds in red) of all of the Korean H7 viruses, and asterisks denote the Korean H7 viruses isolated in the present study. Phylogenetic trees were constructed using the maximum likelihood method with general time-reversible model with invariant sites and 4 gamma-distributed heterogeneous substitution rates (GTR+ I + Γ4 model) and 100 bootstrap replications (H7 I = 0.285 α = 1.092; N9 I = 0.416 α = 1.452; N7 I = 0.411 α = 1.528; N3 I = 0.269 α = 0.858; N8 I = 0.371 α = 1.162; N2 I = 0.417 α = 1.590; N6 I = 0.309 α = 0.962) in PhyML 3.0 [11]. Statistical support for the phylogenies was assessed by the approximate likelihood test using a Shimodaira-Hasegawa-like procedure in PhyML 3.0. The topology of trees was visualized in FigTree 1.4. Viruses from different hosts are indicated by: wild birds, green; poultry, orange; human, pink.

**Figure 5:**
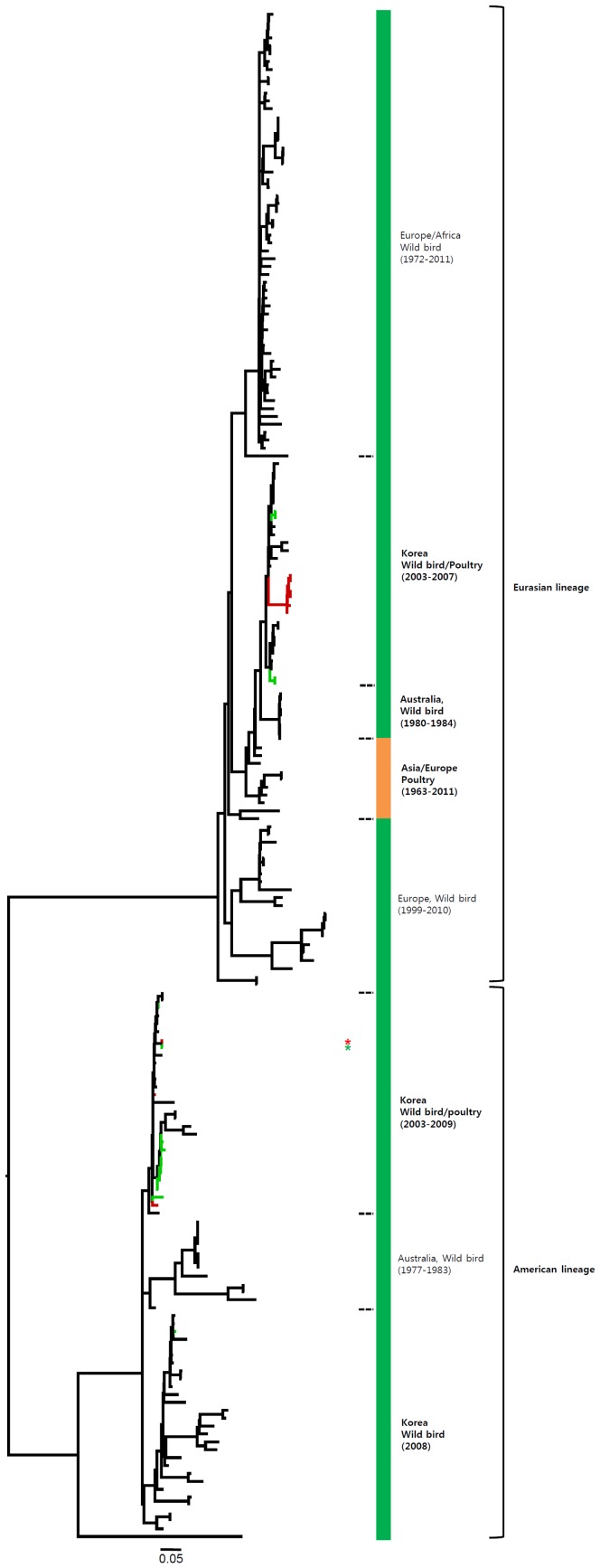
Phylogenies of N8 (*n* = 194) genes. Tip and branch colors represent host origin (wild birds in green, domestic birds in red) of all of the Korean H7 viruses, and asterisks denote the Korean H7 viruses isolated in the present study. Phylogenetic trees were constructed using the maximum likelihood method with general time-reversible model with invariant sites and 4 gamma-distributed heterogeneous substitution rates (GTR+ I + Γ4 model) and 100 bootstrap replications (H7 I = 0.285 α = 1.092; N9 I = 0.416 α = 1.452; N7 I = 0.411 α = 1.528; N3 I = 0.269 α = 0.858; N8 I = 0.371 α = 1.162; N2 I = 0.417 α = 1.590; N6 I = 0.309 α = 0.962) in PhyML 3.0 [11]. Statistical support for the phylogenies was assessed by the approximate likelihood test using a Shimodaira-Hasegawa-like procedure in PhyML 3.0. The topology of trees was visualized in FigTree 1.4. Viruses from different hosts are indicated by: wild birds, green; poultry, orange; human, pink.

**Figure 6:**
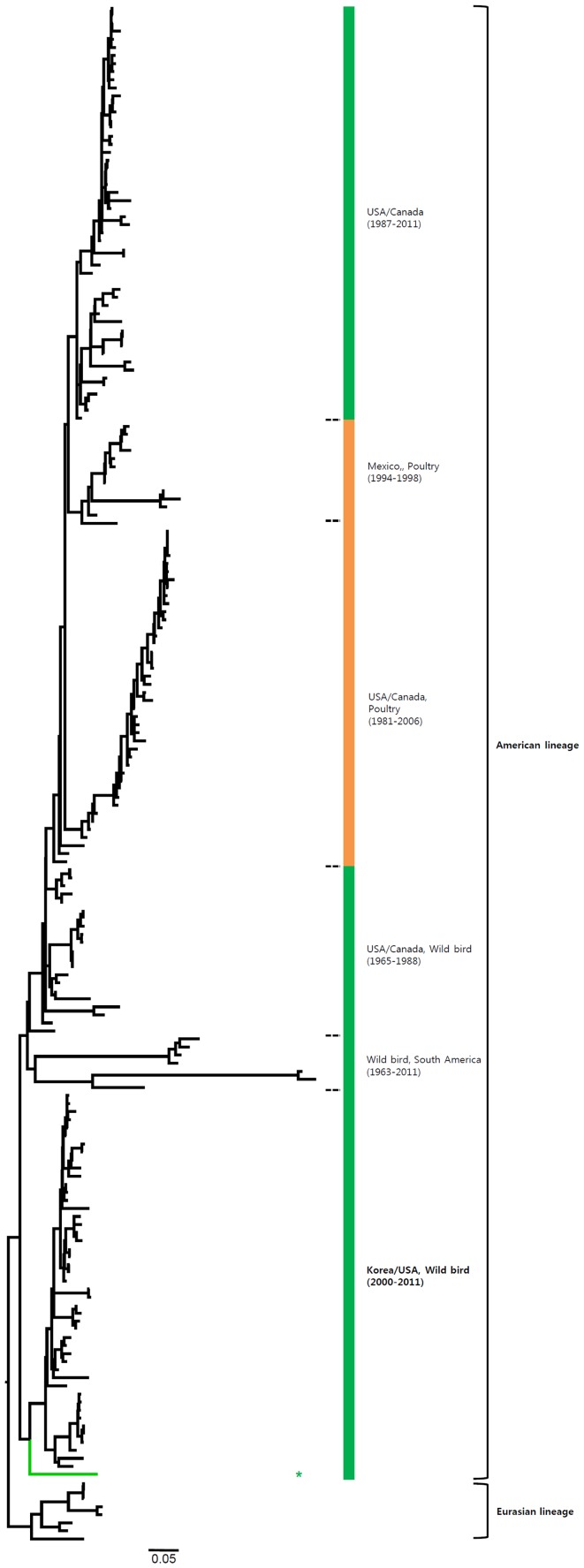
Phylogenies of N2 (*n* = 191) genes. Tip and branch colors represent host origin (wild birds in green, domestic birds in red) of all of the Korean H7 viruses, and asterisks denote the Korean H7 viruses isolated in the present study. Phylogenetic trees were constructed using the maximum likelihood method with general time-reversible model with invariant sites and 4 gamma-distributed heterogeneous substitution rates (GTR+ I + Γ4 model) and 100 bootstrap replications (H7 I = 0.285 α = 1.092; N9 I = 0.416 α = 1.452; N7 I = 0.411 α = 1.528; N3 I = 0.269 α = 0.858; N8 I = 0.371 α = 1.162; N2 I = 0.417 α = 1.590; N6 I = 0.309 α = 0.962) in PhyML 3.0 [11]. Statistical support for the phylogenies was assessed by the approximate likelihood test using a Shimodaira-Hasegawa-like procedure in PhyML 3.0. The topology of trees was visualized in FigTree 1.4. Viruses from different hosts are indicated by: wild birds, green; poultry, orange; human, pink.

**Figure 7:**
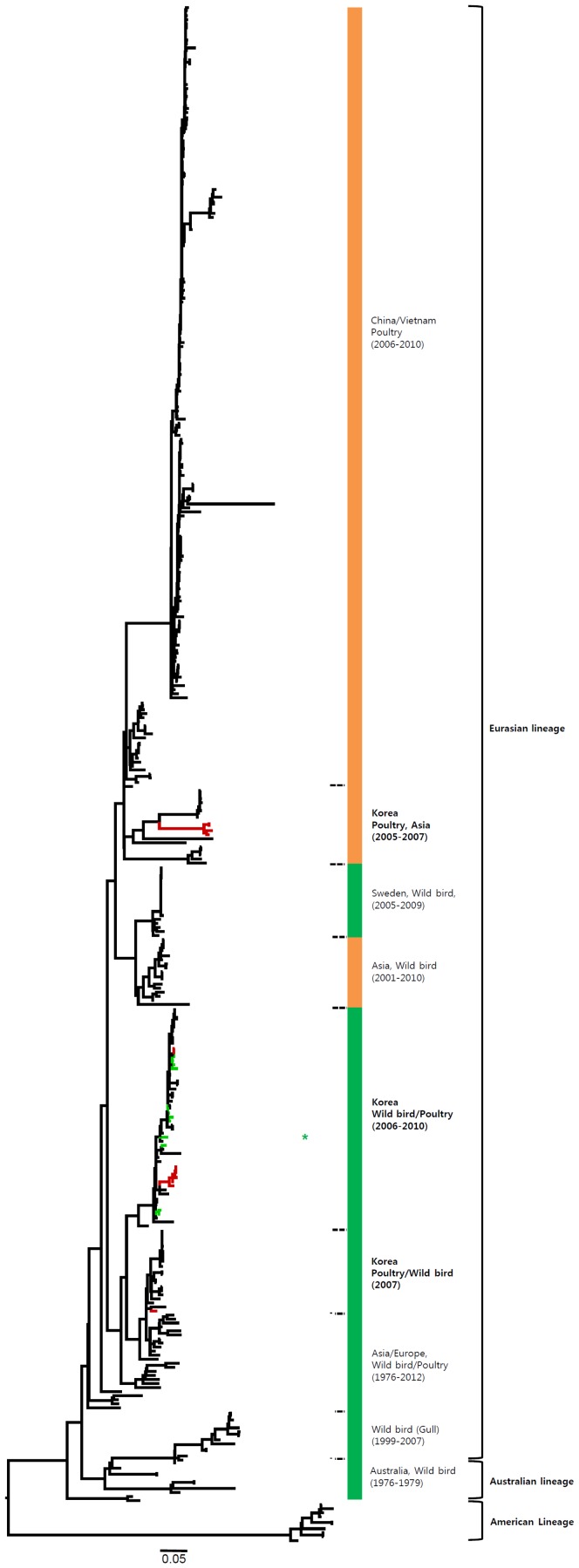
Phylogenies of N6 (*n* = 381) genes. Tip and branch colors represent host origin (wild birds in green, domestic birds in red) of all of the Korean H7 viruses, and asterisks denote the Korean H7 viruses isolated in the present study. Phylogenetic trees were constructed using the maximum likelihood method with general time-reversible model with invariant sites and 4 gamma-distributed heterogeneous substitution rates (GTR+ I + Γ4 model) and 100 bootstrap replications (H7 I = 0.285 α = 1.092; N9 I = 0.416 α = 1.452; N7 I = 0.411 α = 1.528; N3 I = 0.269 α = 0.858; N8 I = 0.371 α = 1.162; N2 I = 0.417 α = 1.590; N6 I = 0.309 α = 0.962) in PhyML 3.0 [11]. Statistical support for the phylogenies was assessed by the approximate likelihood test using a Shimodaira-Hasegawa-like procedure in PhyML 3.0. The topology of trees was visualized in FigTree 1.4. Viruses from different hosts are indicated by: wild birds, green; poultry, orange; human, pink.

Most of the NA genes of the Korean H7 viruses belonged to the Eurasian lineage. However, one virus (A/mallard/Korea/GJ62/2007 (H7N2)) clustered with the American lineage in the N2 gene and fourteen viruses including one virus (A/duck/Korea/GJ56/2007 (H7N8)) in our study clustered with the American lineage in the N8 gene (Figures 2–7 and Figures S1b–g). Like the HA gene, the NA genes of the Korean H7 viruses showed genetic diversity within the Eurasian lineage and most of them were isolated from wild birds, although the viruses were sometimes detected in poultry and humans.

**Figure 8:**
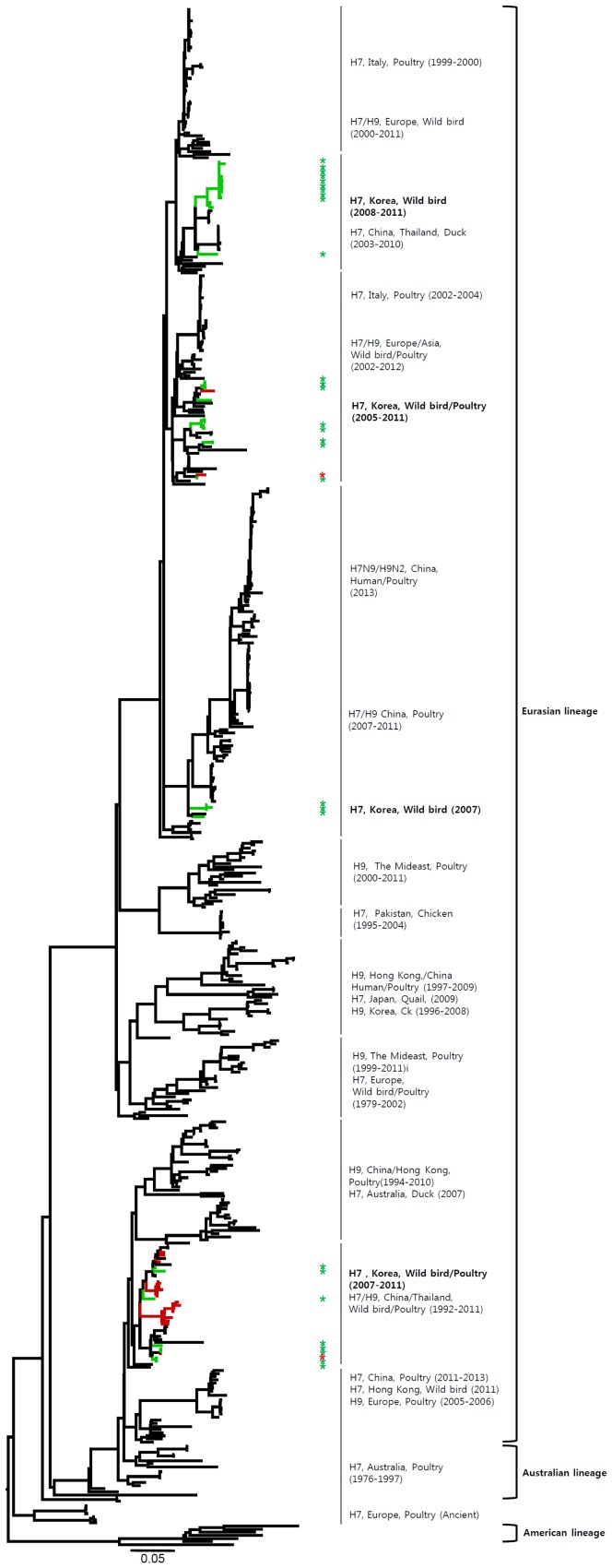
Phylogenies of PB2 (*n* = 495) genes. Tip and branch colors represent host origin (wild birds in green, domestic birds in red) of all of the Korean H7 viruses, and asterisks denote the Korean H7 viruses isolated in the present study. Phylogenetic trees were constructed using the maximum likelihood method with a general time-reversible model with invariant sites and 4 gamma-distributed heterogeneous substitution rates (GTR+ I + Γ4 model) and 100 bootstrap replications (PB2 I = 0.308 α = 0.749; PB1 I = 0.377 α = 0.899; PA I = 0.320 α = 0.773; NP 0.409 α = 0.874; M I = 0.146 α = 0.435; NS I = 0.161 α = 0.768) in PhyML 3.0 (Guindon et al., 2010). Statistical support for the phylogenies was assessed by the approximate likelihood test using a Shimodaira-Hasegawa-like procedure in PhyML 3.0. The topology of trees was visualized in FigTree 1.4.

**Figure 9:**
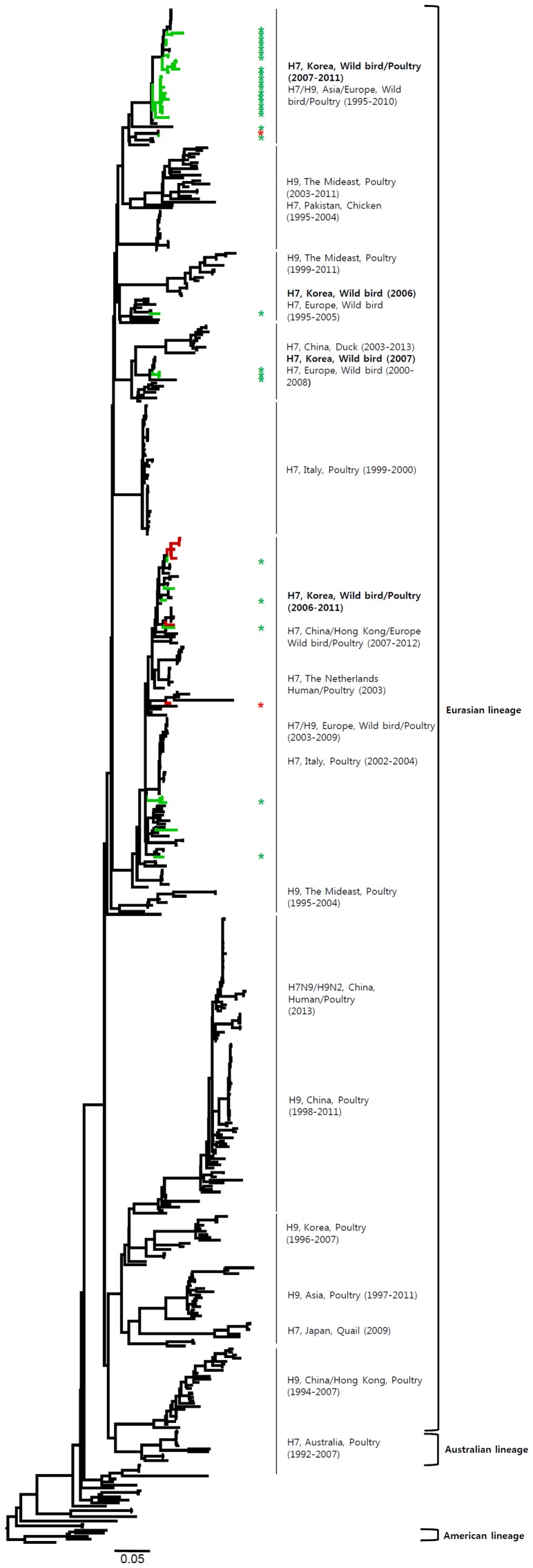
Phylogenies of PB1 (*n* = 509) genes. Tip and branch colors represent host origin (wild birds in green, domestic birds in red) of all of the Korean H7 viruses, and asterisks denote the Korean H7 viruses isolated in the present study. Phylogenetic trees were constructed using the maximum likelihood method with a general time-reversible model with invariant sites and 4 gamma-distributed heterogeneous substitution rates (GTR+ I + Γ4 model) and 100 bootstrap replications (PB2 I = 0.308 α = 0.749; PB1 I = 0.377 α = 0.899; PA I = 0.320 α = 0.773; NP 0.409 α = 0.874; M I = 0.146 α = 0.435; NS I = 0.161 α = 0.768) in PhyML 3.0 (Guindon et al., 2010). Statistical support for the phylogenies was assessed by the approximate likelihood test using a Shimodaira-Hasegawa-like procedure in PhyML 3.0. The topology of trees was visualized in FigTree 1.4.

**Figure 10:**
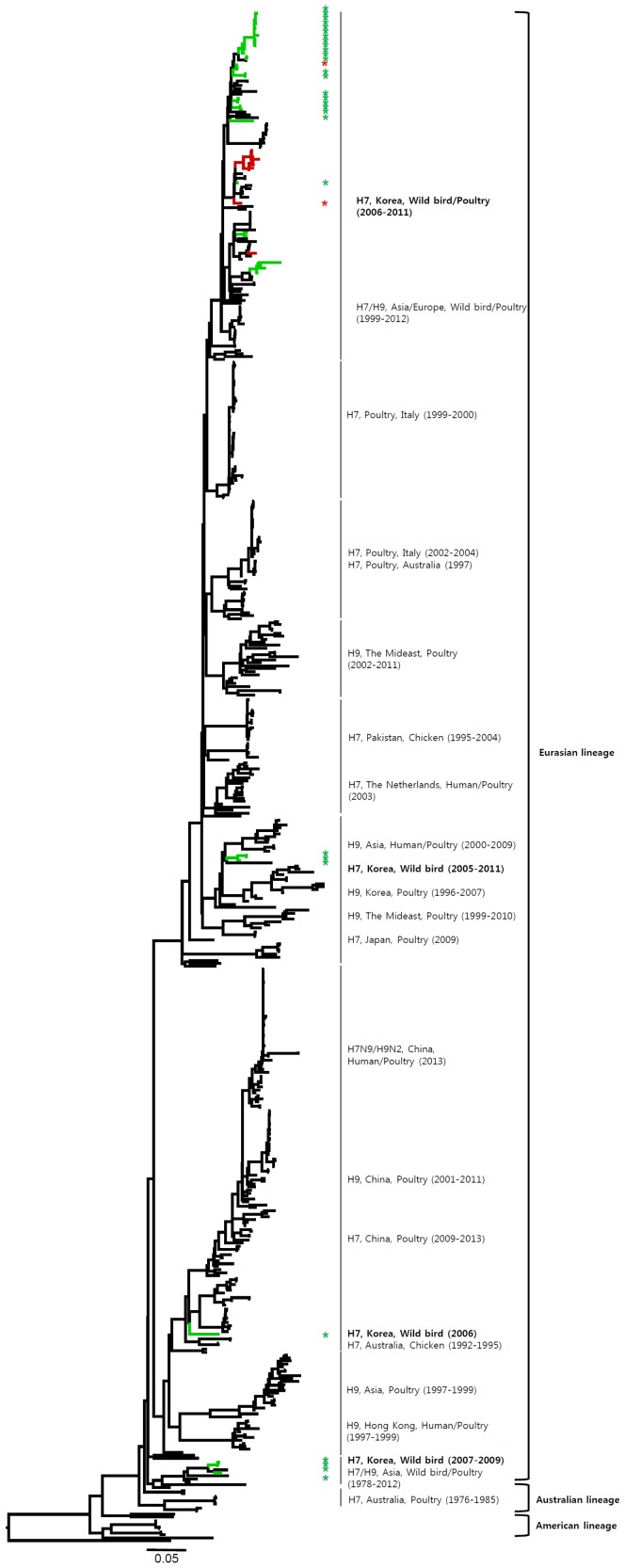
Phylogenies of PA (*n* = 520) genes. Tip and branch colors represent host origin (wild birds in green, domestic birds in red) of all of the Korean H7 viruses, and asterisks denote the Korean H7 viruses isolated in the present study. Phylogenetic trees were constructed using the maximum likelihood method with a general time-reversible model with invariant sites and 4 gamma-distributed heterogeneous substitution rates (GTR+ I + Γ4 model) and 100 bootstrap replications (PB2 I = 0.308 α = 0.749; PB1 I = 0.377 α = 0.899; PA I = 0.320 α = 0.773; NP 0.409 α = 0.874; M I = 0.146 α = 0.435; NS I = 0.161 α = 0.768) in PhyML 3.0 (Guindon et al., 2010). Statistical support for the phylogenies was assessed by the approximate likelihood test using a Shimodaira-Hasegawa-like procedure in PhyML 3.0. The topology of trees was visualized in FigTree 1.4.

**Figure 11:**
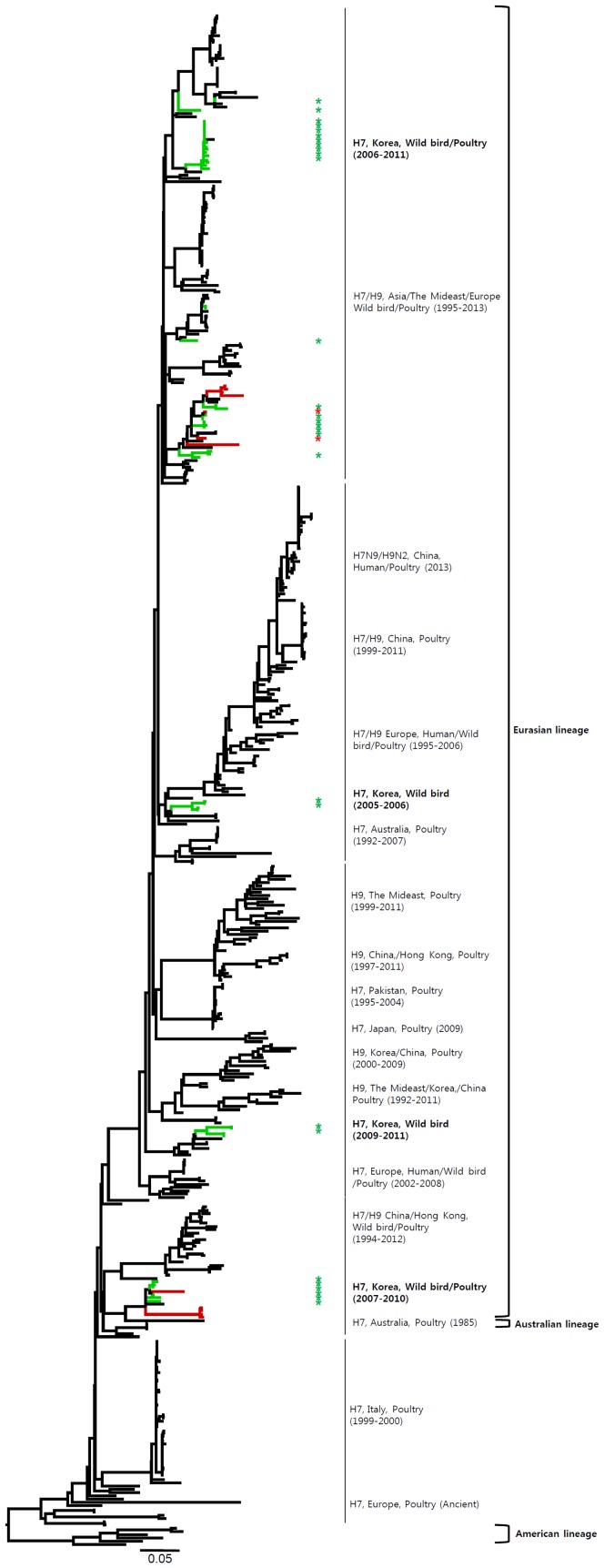
Phylogenies of NP (*n* = 472) genes. Tip and branch colors represent host origin (wild birds in green, domestic birds in red) of all of the Korean H7 viruses, and asterisks denote the Korean H7 viruses isolated in the present study. Phylogenetic trees were constructed using the maximum likelihood method with a general time-reversible model with invariant sites and 4 gamma-distributed heterogeneous substitution rates (GTR+ I + Γ4 model) and 100 bootstrap replications (PB2 I = 0.308 α = 0.749; PB1 I = 0.377 α = 0.899; PA I = 0.320 α = 0.773; NP 0.409 α = 0.874; M I = 0.146 α = 0.435; NS I = 0.161 α = 0.768) in PhyML 3.0 (Guindon et al., 2010). Statistical support for the phylogenies was assessed by the approximate likelihood test using a Shimodaira-Hasegawa-like procedure in PhyML 3.0. The topology of trees was visualized in FigTree 1.4.

**Figure 12:**
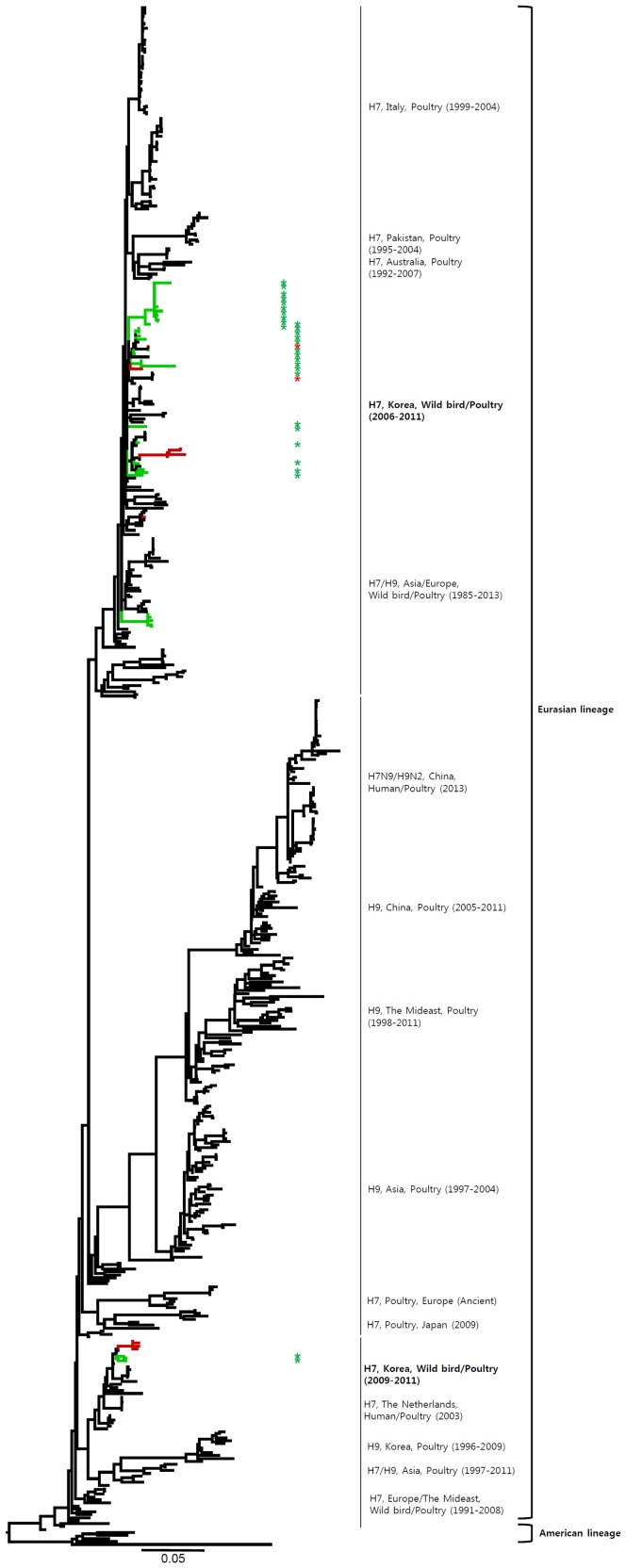
Phylogenies of M (*n* = 520) genes. Tip and branch colors represent host origin (wild birds in green, domestic birds in red) of all of the Korean H7 viruses, and asterisks denote the Korean H7 viruses isolated in the present study. Phylogenetic trees were constructed using the maximum likelihood method with a general time-reversible model with invariant sites and 4 gamma-distributed heterogeneous substitution rates (GTR+ I + Γ4 model) and 100 bootstrap replications (PB2 I = 0.308 α = 0.749; PB1 I = 0.377 α = 0.899; PA I = 0.320 α = 0.773; NP 0.409 α = 0.874; M I = 0.146 α = 0.435; NS I = 0.161 α = 0.768) in PhyML 3.0 (Guindon et al., 2010). Statistical support for the phylogenies was assessed by the approximate likelihood test using a Shimodaira-Hasegawa-like procedure in PhyML 3.0. The topology of trees was visualized in FigTree 1.4.

**Figure 13:**
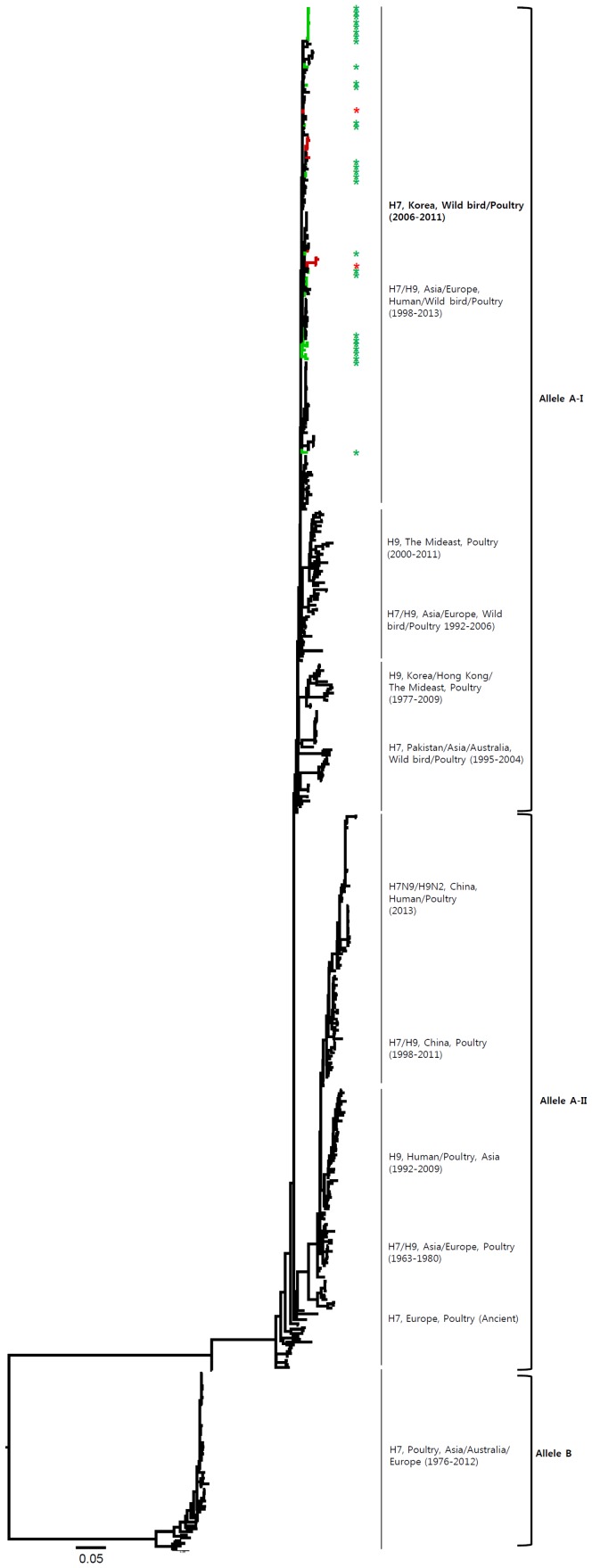
Phylogenies of NS (*n* = 545) genes. Tip and branch colors represent host origin (wild birds in green, domestic birds in red) of all of the Korean H7 viruses, and asterisks denote the Korean H7 viruses isolated in the present study. Phylogenetic trees were constructed using the maximum likelihood method with a general time-reversible model with invariant sites and 4 gamma-distributed heterogeneous substitution rates (GTR+ I + Γ4 model) and 100 bootstrap replications (PB2 I = 0.308 α = 0.749; PB1 I = 0.377 α = 0.899; PA I = 0.320 α = 0.773; NP 0.409 α = 0.874; M I = 0.146 α = 0.435; NS I = 0.161 α = 0.768) in PhyML 3.0 (Guindon et al., 2010). Statistical support for the phylogenies was assessed by the approximate likelihood test using a Shimodaira-Hasegawa-like procedure in PhyML 3.0. The topology of trees was visualized in FigTree 1.4.

Phylogenetic analysis of internal genes indicated that the H7 viruses of the Eurasian lineage were composed of various sublineages, which showed genetic relationships between wild birds and poultry, dependent on regions and periods of isolation of the H7 viruses (Figures 8–13 and Figures S2a–f). The internal genes of H7N9 viruses of China are highly related to the H9N2 viruses in poultry [21], [35], [39]. Therefore, in addition to the H7 viruses, we also included the H9 viruses in our analysis of internal genes in the present study. With the exception of NS, all of the internal genes of H7 Korean viruses had two to four distinct sublineages, which were related to the H7 or H9 viruses previously isolated from various wild birds and poultry in Asia and Europe. Although the PB2, PA, and NP genes of some Korean viruses clustered with the H7N9 viruses from humans and poultry in China, showing 86.1–97.3%, 91.8–95.6%, and 90.0–94.8% similarity, respectively, all of the internal genes of the H7N9 viruses shared the highest similarity with H9N2 viruses (96.3–99.2%) circulating in poultry in eastern China (Figures 8–13 and Figures S2a–f). The NS genes were composed of two major lineages, allele A and allele B. The viruses mostly belonged to the allele A lineage, which could be divided into two distinct sublineages, namely, allele A-I and allele A-II. Allele A-I was composed of H7 and H9 viruses from wild birds and poultry in Asia and Europe, including Korea, whereas allele A-II included the H7N9 and H9N2 viruses from humans and poultry in China isolated in 2013 (Figure 13 and Figure S2f).

Two viruses originating in domestic ducks (A/duck/Korea/BC10/2007 and A/duck/Korea/GJ56/2007) in our study had the same gene constellations in all segment genes as viruses originating in wild birds. A/duck/Korea/BC10/2007 and A/duck/Korea/GJ56/2007 were detected from broiler ducks and domesticated mallard ducks of living on farms, respectively. A/duck/Korea/BC10/2007 was closely related to those from bean goose in 2008 (A/bean goose/Korea/SH20-17/2008(H7N3)) with 93.6–98.6% (PB2 93.6%, PB1 96.5%, PA 96.3%, HA 97.0%, NP 95.8%, NA 98.0%, M 98.6%, and NS 98.4%) and 96.8–99.5% (PB2 99.2%, PB1 99.4%, PA 99.0%, HA 97.1%, NP 99.3%, NA 98.9%, M 98.8%, and NS 99.5%) similarity at the nucleotide and amino acid level, respectively. A/duck/Korea/GJ56/2007 was closely related to those from mallard in 2007 (A/mallard/Korea/GJ63/2007(H7N8)) with 99.7–100% (PB2 99.9%, PB1 100%, PA 99.9%, HA 99.7%, NP 99.7%, NA 99.8%, M 100%, and NS 100%) and 99.7–100% (PB2 99.8%, PB1 100, PA 100%, HA 99.8%, NP 99.7%, NA 99.7%, M 100%, and NS 100%) similarity at the nucleotide and amino acid level, respectively.

### Molecular characterization

The Korean H7 isolates analyzed in this study had various motifs, PEIPKGR, PEPPKGR, PETPKGR, PEIPKRR, and PELPKGR, all with one or two amino acid R (arginine) residues at the HA cleavage site, which have been associated with low pathogenic effects in poultry. The H7N9 viruses of China also had the PEIPKGR motif (Table 1). The Korean H7 isolates had avian-type receptors (Q226 and G228) and, moreover, they had no amino acid substitutions of E627 in PB2 and I368 in PB1. By contrast, six of the H7N9 viruses had the substitution mutations Q226L or Q226I, which could increase binding to human-type receptors. In addition, the substitutions E627K and I368V were also found. The E627K substitution may serve to increase mammalian host adaptation and the I368V substitution has been correlated with H5 virus transmission among ferrets. The Korean H7 viruses had no deletions of the five key amino acids at positions 69–73 in their NA stalk regions and no C-terminal deletion of the PDZ ligand in NS1. The H7N9 viruses, on the other hand, were deleted in the NA stalk region, a mutation that has been reported to occur upon virus adaptation to terrestrial birds. In addition, the H7N9 viruses contained a deletion of amino acids 218–230 in NS1, which has been associated with potential adaptation to a non-avian host. The M2 protein of all of the Korean viruses maintained the residue S31, whereas all of the H7N9 viruses had mutated to S31N, indicating resistance to amantadine and rimantadine.

### Pathogenicity of the Korean H7 viruses in domestic ducks and mice

To assess the pathogenicity of selected H7 IAVs in domestic ducks and mice, five different viruses (A/duck/Korea/BC10/2007, A/duck/Korea/GJ56/2007, A/wild duck/Korea/MHC35-41/2011, A/wild duck/Korea/CSM27-12/2009, and A/common teal/Korea/MHC5-8/2009) were intranasally inoculated into 2-week-old domestic ducks (10^6.5^ EID_50_/100 ul) and 6-week-old BALB/c mice (10^6.5^ EID_50_/50 ul). None of the H7 IAVs tested induced clinical signs in inoculated domestic ducks or mice. In domestic ducks, all five viruses were recovered in OP swabs at low titers (10 ^0.7–1.3^EID_50_/50 µl). In addition, most of the IAVs were rarely seen in cloacal swabs, up to 10 dpi. Likewise, no IAVs had replicated in any of the tissue samples tested (brain, trachea, lung, cecal tonsil, kidney, and spleen). In the lung samples from mice, however, all five viruses had replicated well, up to 7–10 dpi (10 ^0.7–4.3^EID_50_/50 µl) (Table 2). These results indicated that all five viruses did not replicate well in domestic ducks, but could replicate well in mammals without prior adaptation.

**Table 2:** Pathotyping and replication of the selected H7 viruses in domestic ducks and mice.

Virus	dpi	Domestic ducks									Mice
		No. of positive/total (virus isolation titer (log_10_ EID_50_/0.1 ml))*								Sero-conversion	No. of positive/total
		OP	CL	Brain	Tra	Lung	CT	Kid	SPL		Lung
A/duck/Korea/BC10/2007	1	5/9 (1.3)	1/9							0/5	1/2
(H7N3)	3	3/9 (0.7)	0/9	0/2	0/2	0/2	0/2	0/2	0/2		0/2
	5	0/7	0/7								2/2 (3.7)
	7	0/7	0/7	0/2	0/2	0/2	0/2	0/2	0/2		1/2 (2.7)
	10	0/5	0/5								0/2
A/duck/Korea/GJ56/2007	1	8/9 (0.9)	0/9							0/5	2/2 (1.7)
(H7N8)	3	1/9 (0.7)	0/9	0/2	0/2	0/2	0/2	0/2	0/2		0/2
	5	0/7	0/7								1/2 (0.7)
	7	0/7	0/7	0/2	0/2	0/2	0/2	0/2	0/2		2/2 (3.9)
	10	0/5	0/5								1/2
A/wild duck/Korea/MHC35-41/2011	1	6/9 (0.7)	0/9							0/5	1/2
(H7N9)	3	2/9	0/9	0/2	0/2	0/2	0/2	0/2	0/2		2/2 (2.5)
	5	0/7	0/7								2/2 (3.1)
	7	0/7	0/7	0/2	0/2	0/2	0/2	0/2	0/2		2/2 (4.1)
	10	0/5	0/5								1/2
A/wild duck/Korea/CSM27-12/2009	1	4/9	0/9							0/5	2/2 (1.7)
(H7N6)	3	1/9	0/9	0/2	0/2	0/2	0/2	0/2	0/2		1/2 (3.1)
	5	0/7	0/7								2/2 (4.3)
	7	0/7	0/7	0/2	0/2	0/2	0/2	0/2	0/2		1/2 (2.3)
	10	0/5	0/5								0/2
A/common teal/Korea/MHC5-8/2009	1	4/9	1/9							0/5	2/2 (1.1)
(H7N7)	3	1/9	0/9	0/2	0/2	0/2	0/2	0/2	0/2		1/2 (3.9)
	5	2/7	0/7								2/2 (3.5)
	7	0/7	0/7	0/2	0/2	0/2	0/2	0/2	0/2		1/2
	10	0/5	0/5								0/2

*Number of virus detected animals/number of virus inoculated animals. Virus titers inoculated intranasally with 10^6.5^EID_50_/0.1 ml of the virus.

### Antigenic analysis of the Korean H7 viruses

The HI test was used to analyze the antigenic relationships among the selected H7 viruses using antisera in SPF chickens. The antisera against these viruses cross-reacted well together (*r*-value 0.125–0.5), providing evidence of the antigenic similarity among the Korean H7 IAVs (Table 3).

**Table 3:** Antigenic analysis of H7 viruses isolated in Korea.

Virus antigen	Duck antisera				
	BC10/07	GJ56/07	MHC35-41/11	CSM27-12/09	MHC5-8/09
A/duck/Korea/BC10/2007	**512** *	128	128	128	512
		(0.125)**	(0.25)	(0.25)	(0.125)
A/duck/Korea/GJ56/2007	128	**128**	128	128	512
			(0.25)	(0.5)	(0.25)
A/wild duck/Korea/MHC35-41/2011	512	128	**256**	128	512
				(0.125)	(0.125)
A/wild duck/Korea/CSM27-12/2009	512	256	128	**256**	1024
					(0.125)
A/common teal/Korea/MHC5-8/2009	128	64	64	32	**512**

*Values shown are HI titers. The titer of the homologous antigen group is shown in bold.

**r-value = (r^1^×r^2^)1/2, r^1^ = heterologous titer with virus 2/homologous titer with virus 1.

r^2^ = heterologous titer with virus 1/homologous titer with virus.

## Discussion

Due to the capability of LPAI H5 and H7 viruses to mutate into highly pathogenic forms, infections of commercial poultry with any viruses of the H5 or H7 subtype, regardless of their pathogenicity, are nowadays classified as “notifiable avian influenza (NAI)”, and initiate official control measures [45]. Commercial poultry includes all domesticated birds, including backyard poultry and farm-based poultry, used for the production of meat or eggs for consumption, for the production of other commercial products, for restocking supplies of game, or for breeding, as well as fighting cocks used for any purpose [44]. Monitoring of H5 and H7 viruses from poultry through surveillance programs is needed to eliminate these viruses at an early stage and to thereby prevent the emergence of novel variant viruses.

We isolated 31 H7 subtypes during a nationwide surveillance program between January 2006 and March 2011. The major subtypes of the H7 IAVs were H7N7 (39.7%) and H7N9 (35.5%) in wild birds, similar to previous surveys of low pathogenic avian influenza viruses (LPAIVs) in European and Korean wild waterfowl [23], [25], [38], [40]. Two of the viruses isolated in the current study were extracted from domestic ducks in 2007, and the others were taken from migratory birds at different points over the duration of the surveillance program.

Phylogenetic analysis of the HA genes of the Korean H7 viruses revealed a genetically diverse population that could be divided into three sublineages (Korea-I, II, and III). The Korean H7 viruses from poultry belonged to Korea-I, which was highly related to those from wild birds in Korea. Interestingly, two viruses originating in domestic ducks (A/duck/Korea/BC10/2007 and A/duck/Korea/GJ56/2007) in Korea had the same gene constellations in all segment genes as viruses originating in wild birds (data not shown). These results suggest that the two domestic Korean viruses were transferred directly from wild birds through at least two independent introductions.

The HA gene of the H7N9 viruses isolated from humans in China originated from poultry sold in live bird markets. These viruses were phylogenetically close to four H7N3 viruses (94.5–95.4%) isolated from ducks in Zhejiang in 2010–2012 (Figure 1 and Figure S1a). Before the H7N9 outbreaks, H7N2 and H7N3 subtypes of viruses have circulated in poultry in China for several years [12], [34]. In addition, since 1996, H9N2 influenza viruses have been widespread, circulating in chickens and other poultry in the eastern China region, such as Shanghai, Jiangsu, Zhejiang, Anhui, Shangdong, and Jiangxi [46]. Previous studies have demonstrated that the internal genes of H7N9 viruses clustered with chicken H9N2 viruses in the vicinity of Shanghai in 2010–2012, suggesting that H9N2 viruses in chickens of eastern China may be possible donors of novel H7N9 internal genes [4], [21], [35]. It has been suggested that long-term maintenance of these genes could have been at the root of the emergence of the H7N9 virus [31], [35].

Phylogenetic analysis of internal genes revealed that all of the internal genes of H7 Korean viruses, except for the NS gene, showed genetic diversity, with two to four distinct sublineages that were related to the H7 or H9 viruses from various wild birds and poultry in Asia and Europe. The PB2, PA, and NP genes of some Korean H7 viruses clustered with those of the H7N9 or H9N2 viruses from humans and poultry in China in 2013, with 86.1–97.3%, 91.8–95.6%, and 90.0–94.8% similarity, respectively. However, all of the internal genes of H7N9 viruses shared the highest similarity with H9N2 viruses (96.3–99.2%) circulating in poultry in eastern China rather than Korean H7 viruses (Figures 8–13 and Figures S2a–f).

Most of the NA genes of the Korean H7 viruses belonged to the Eurasian lineage, whereas one of the N2 viruses and some of the N8 viruses belonged to the American lineage (Figures 5, 6 and Figures S1e, S1f). This finding indicates that, through overlapping flyways, IAVs have become mixed among different migratory bird species, driving the spread of the virus over long distances between continents [41]. The N9 gene of the Korean H7 viruses from wild birds was related to H7N9 viruses causing human infections (93.8–97.2%), and may have originated from IAV carried by wild birds.

None of the Korean H7 isolates had deletions in their NA stalk regions, whereas all H7N9 viruses of China were deleted in the stalk region of NA residues 69 to 73 in both human- and poultry-origin viruses. The Korean H7 isolates and the H7N9 viruses showed distinct differences in the receptor binding sites. None of the Korean H7 viruses had substitutions at position 226 of the HA1, whereas substitution Q226L or Q226I in the HA gene was found in six of the H7N9 viruses in China. Genetic adaptation after introduction of the virus to domestic ducks in Korea was not detected in the isolated viruses and sequenced gene segments. This finding stresses the necessity for active surveillance in poultry, even in populations that exhibit no clinical signs.

The virulence of influenza virus is a multigenic trait [2], [5], [10]. Lysine at position 627 of the polymerase PB2 protein and deletion of 218–230 in NS1 are essential for the efficient replication of avian influenza viruses in mammals and potential adaptation to non-avian hosts, respectively [14], [20]. The Korean H7 isolates contained E627 in PB2 and no deletion in NS1, whereas all four H7N9 viruses were substituted from glutamic acid to lysine in position 627 of PB2 and the PDZ domain-binding motif was deleted. In addition, unlike the Korean H7 isolates, the H7N9 viruses contained other mutations in key amino acids; PB1–368V (except A/Shanghai/1/13), PA–100A, PA–356R, and PA–409N, which may be associated with increased virulence and bird-to-human transmissibility [21]. H7N9 viruses in China showed multiple amino acid substitutions in the functional domains of the viral proteins, which is rarely seen in natural reservoirs. This finding suggests that H7N9 viruses may be circulating and mutating in non-natural reservoirs such as gallinaceous birds.

Antiviral compounds are the first line of defense against influenza viruses until vaccines become available [21]. The M2 protein showed no substitution at position 31 in the Korean strains, whereas all H7N9 viruses contained the S31N substitution, which is correlated with resistance to the M2 channel blockers amantadine and rimantadine [15], [36]. Fortunately, with the exception of the A/Shanghai/1/13 virus, none of the Korean H7 viruses and H7N9 viruses showed any evidence of the R294K substitution in NA, which would indicate resistance to oseltamivir.

To determine the pathogenicity of selected H7 viruses, we experimented on Pekin ducks (*Anas platyrhynchos domesticus*). In our study, none of the H7 viruses tested induced clinical signs in domestic ducks. The H7 viruses showed no efficient replication in the ducks, according to the results from OP swabs (low titers) and cloacal swabs and major tissue samples (Table 2). In the past, LPAI viruses in aquatic birds were found to preferentially replicate in intestinal tissue [17], [24], [42]. However, similar to our study, other studies in domestic ducks which investigated shedding of LPAI H7N3, H3, and H4 viruses that originated from wild birds found that they replicated only in OP swabs, to low titer [6], [7], [22]. Our results may have potential problems such as the inoculation technique or lower the infectious does although it has limit to estimate exactly as the control group was not included in our study [7].

In mice, used as a mammalian model, all five viruses isolated from domestic ducks and wild birds in Korea efficiently replicated in the lungs. The viruses reached a relatively high titer although there were few mutations in residues associated with increased virulence in mammals (Tables 1 and 2). Our results were consistent with other reports that H7 viruses from chickens and wild birds in northern China and the United States replicated well in mice without prior adaptation [8], [34].

In conclusion, the Korean H7 viruses from wild birds showed genetic diversity and Korean H7 viruses from poultry were highly related to those of wild birds in Korea. Interestingly, two viruses originating in domestic ducks (A/duck/Korea/BC10/2007 and /duck/Korea/GJ56/2007) had the same gene constellations in all segment genes as viruses originating in wild birds, whereas the H7N9 viruses could have emerged through long-term maintenance of H7 and H9 viruses in poultry in China. This suggests that wild birds did not carry poultry viruses between Korea and China, but rather, that wild-type H7 viruses were independently introduced several times into poultry in eastern Asia, with no role whatsoever for wild bird migrations between Korea and China.

## Supporting Information

Figure S1 **Phylogenies of seven surface genes: H7 (** ***n*** **= 541) (a), N9 (** ***n*** **= 179) (b), N7 (** ***n*** **= 148) (c), N3 (** ***n*** **= 273) (d), N8 (** ***n*** **= 194) (e), N2 (** ***n*** **= 191) (f), and N6 (** ***n*** **= 381) (g).** Tip and branch colors represent host origin (wild birds in green, domestic birds in red) of all of the Korean H7 viruses. Phylogenetic trees were constructed using the maximum likelihood method with general time-reversible model with invariant sites and 4 gamma-distributed heterogeneous substitution rates (GTR+ I + Γ4 model) and 100 bootstrap replications (H7 I = 0.285 α = 1.092; N9 I = 0.416 α = 1.452; N7 I = 0.411 α = 1.528; N3 I = 0.269 α = 0.858; N8 I = 0.371 α = 1.162; N2 I = 0.417 α = 1.590; N6 I = 0.309 α = 0.962) in PhyML 3.0 [11]. Statistical support for the phylogenies was assessed by the approximate likelihood test using a Shimodaira-Hasegawa-like procedure in PhyML 3.0. The topology of trees was visualized in FigTree 1.4. Viruses from different hosts are indicated by: wild birds, green; poultry, orange; human, pink.
(ZIP)

Figure S2 **Phylogenies of six internal genes: PB2 (** ***n*** **= 495) (a), PB1 (** ***n*** **= 509) (b), PA (** ***n*** **= 520) (c), NP (** ***n*** **= 472) (d), M (** ***n*** **= 520) (e), and NS (** ***n*** **= 545) (f).** Tip and branch colors represent host origin (wild birds in green, domestic birds in red) of all of the Korean H7 viruses. Phylogenetic trees were constructed using the maximum likelihood method with a general time-reversible model with invariant sites and 4 gamma-distributed heterogeneous substitution rates (GTR+ I + Γ4 model) and 100 bootstrap replications (PB2 I = 0.308 α = 0.749; PB1 I = 0.377 α = 0.899; PA I = 0.320 α = 0.773; NP 0.409 α = 0.874; M I = 0.146 α = 0.435; NS I = 0.161 α = 0.768) in PhyML 3.0 (Guindon et al., 2010). Statistical support for the phylogenies was assessed by the approximate likelihood test using a Shimodaira-Hasegawa-like procedure in PhyML 3.0. The topology of trees was visualized in FigTree 1.4.
(ZIP)
