# Supplementary figures and images for: Characterization of H7 Influenza A Virus in Wild and Domestic Birds in Korea
Source: PLoS One. 2014 Apr 28;9(4):e91887. doi: 10.1371/journal.pone.0091887 (PMC4002436; doi:10.1371/journal.pone.0091887)

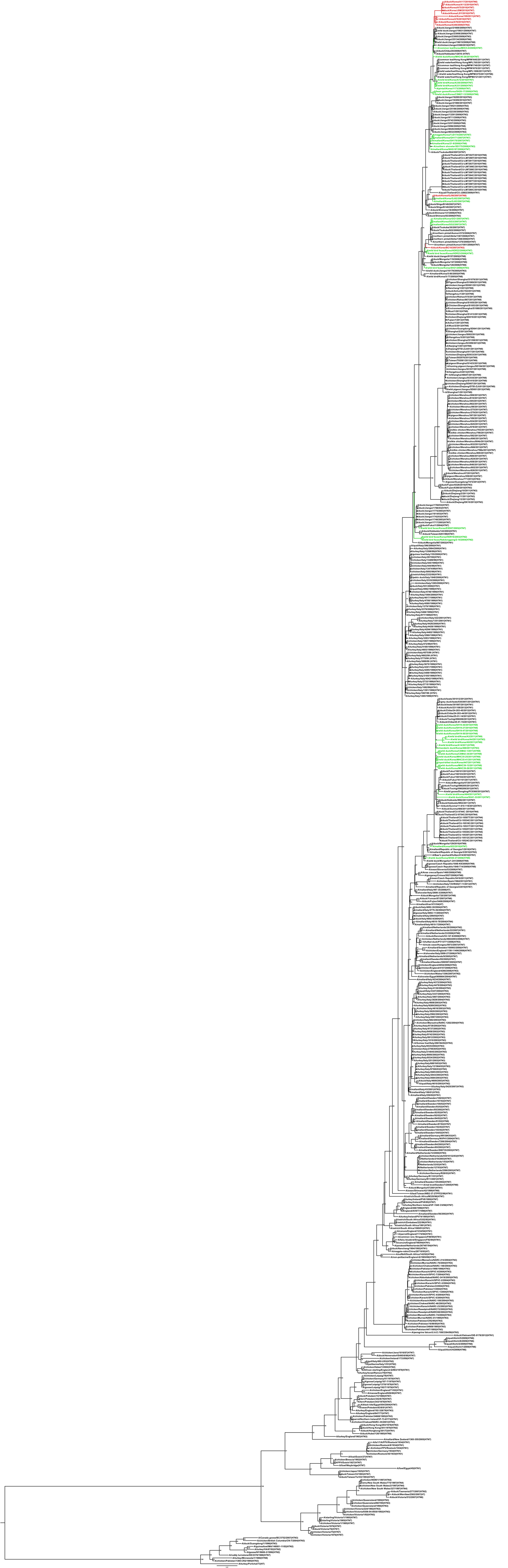

Supplement: Figure S1 — Phylogenies of seven surface genes: H7 ( n = 541) (a), N9 ( n = 179) (b), N7 ( n = 148) (c), N3 ( n = 273) (d), N8 ( n = 194) (e), N2 ( n = 191) (f), and N6 ( n = 381) (g). Tip and branch colors represent host origin (wild birds in green, domestic birds in red) of all of the Korean H7 viruses. Phylogenetic trees were constructed using the maximum likelihood method with general time-reversible model with invariant sites and 4 gamma-distributed heterogeneous substitution rates (GTR+ I + Γ4 model) and 100 bootstrap replications (H7 I = 0.285 α = 1.092; N9 I = 0.416 α = 1.452; N7 I = 0.411 α = 1.528; N3 I = 0.269 α = 0.858; N8 I = 0.371 α = 1.162; N2 I = 0.417 α = 1.590; N6 I = 0.309 α = 0.962) in PhyML 3.0 [11]. Statistical support for the phylogenies was assessed by the approximate likelihood test using a Shimodaira-Hasegawa-like procedure in PhyML 3.0. The topology of trees was visualized in FigTree 1.4. Viruses from different hosts are indicated by: wild birds, green; poultry, orange; human, pink. (ZIP) [file pone.0091887.s001.zip › Figure S1(a) H7.tif]

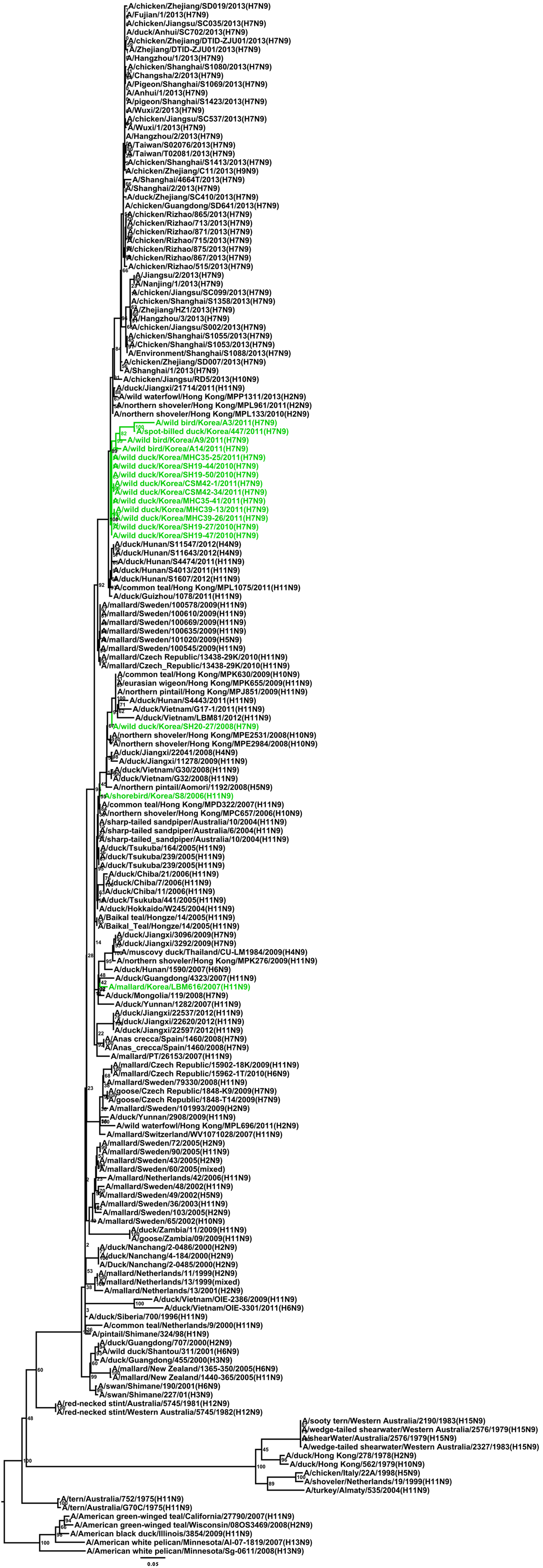

Supplement: Figure S1 — Phylogenies of seven surface genes: H7 ( n = 541) (a), N9 ( n = 179) (b), N7 ( n = 148) (c), N3 ( n = 273) (d), N8 ( n = 194) (e), N2 ( n = 191) (f), and N6 ( n = 381) (g). Tip and branch colors represent host origin (wild birds in green, domestic birds in red) of all of the Korean H7 viruses. Phylogenetic trees were constructed using the maximum likelihood method with general time-reversible model with invariant sites and 4 gamma-distributed heterogeneous substitution rates (GTR+ I + Γ4 model) and 100 bootstrap replications (H7 I = 0.285 α = 1.092; N9 I = 0.416 α = 1.452; N7 I = 0.411 α = 1.528; N3 I = 0.269 α = 0.858; N8 I = 0.371 α = 1.162; N2 I = 0.417 α = 1.590; N6 I = 0.309 α = 0.962) in PhyML 3.0 [11]. Statistical support for the phylogenies was assessed by the approximate likelihood test using a Shimodaira-Hasegawa-like procedure in PhyML 3.0. The topology of trees was visualized in FigTree 1.4. Viruses from different hosts are indicated by: wild birds, green; poultry, orange; human, pink. (ZIP) [file pone.0091887.s001.zip › Figure S1(b) N9.tif]

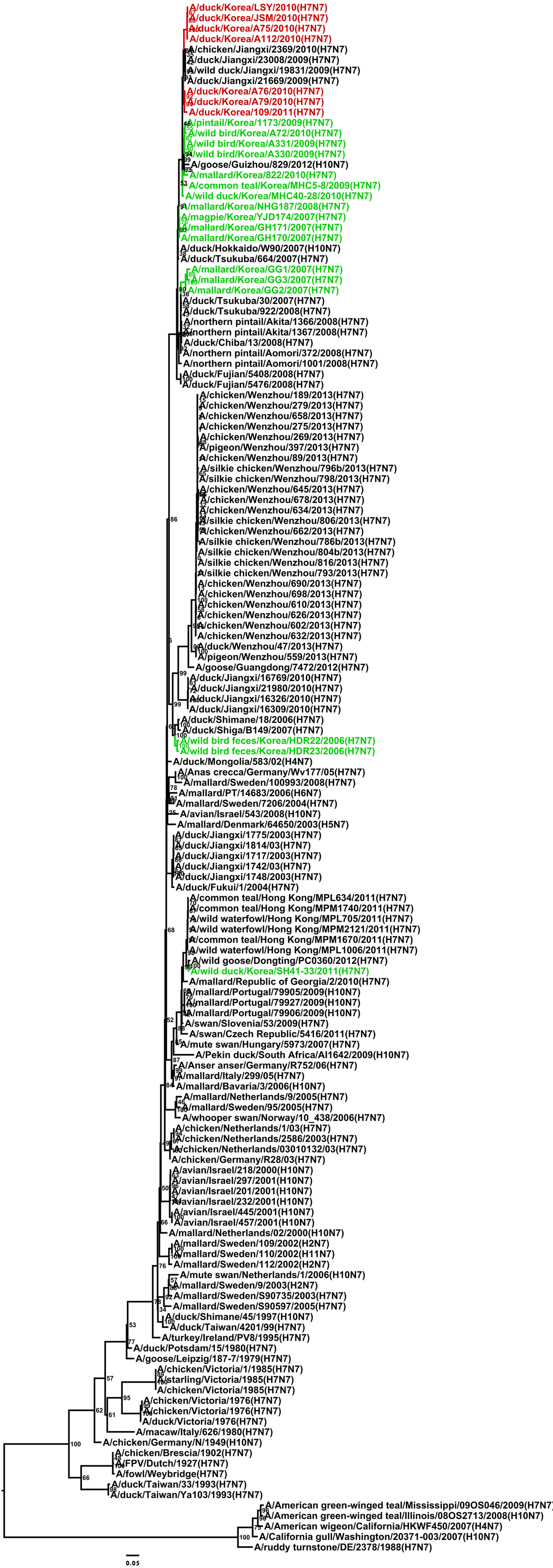

Supplement: Figure S1 — Phylogenies of seven surface genes: H7 ( n = 541) (a), N9 ( n = 179) (b), N7 ( n = 148) (c), N3 ( n = 273) (d), N8 ( n = 194) (e), N2 ( n = 191) (f), and N6 ( n = 381) (g). Tip and branch colors represent host origin (wild birds in green, domestic birds in red) of all of the Korean H7 viruses. Phylogenetic trees were constructed using the maximum likelihood method with general time-reversible model with invariant sites and 4 gamma-distributed heterogeneous substitution rates (GTR+ I + Γ4 model) and 100 bootstrap replications (H7 I = 0.285 α = 1.092; N9 I = 0.416 α = 1.452; N7 I = 0.411 α = 1.528; N3 I = 0.269 α = 0.858; N8 I = 0.371 α = 1.162; N2 I = 0.417 α = 1.590; N6 I = 0.309 α = 0.962) in PhyML 3.0 [11]. Statistical support for the phylogenies was assessed by the approximate likelihood test using a Shimodaira-Hasegawa-like procedure in PhyML 3.0. The topology of trees was visualized in FigTree 1.4. Viruses from different hosts are indicated by: wild birds, green; poultry, orange; human, pink. (ZIP) [file pone.0091887.s001.zip › Figure S1(c) N7.tif]

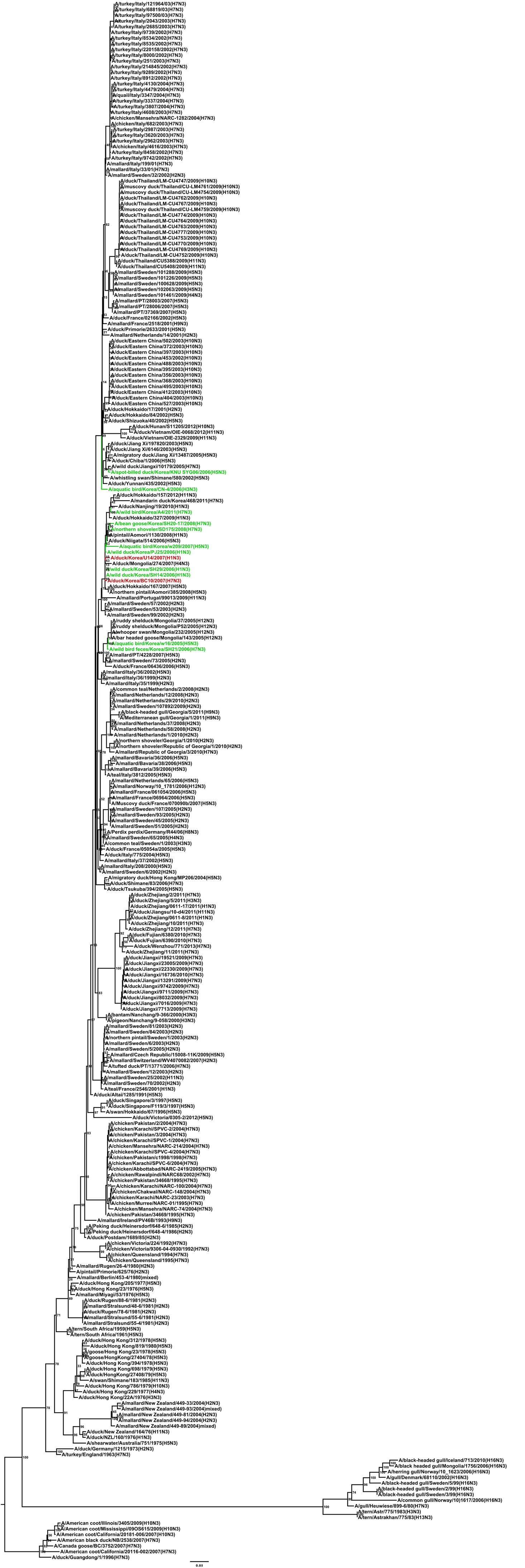

Supplement: Figure S1 — Phylogenies of seven surface genes: H7 ( n = 541) (a), N9 ( n = 179) (b), N7 ( n = 148) (c), N3 ( n = 273) (d), N8 ( n = 194) (e), N2 ( n = 191) (f), and N6 ( n = 381) (g). Tip and branch colors represent host origin (wild birds in green, domestic birds in red) of all of the Korean H7 viruses. Phylogenetic trees were constructed using the maximum likelihood method with general time-reversible model with invariant sites and 4 gamma-distributed heterogeneous substitution rates (GTR+ I + Γ4 model) and 100 bootstrap replications (H7 I = 0.285 α = 1.092; N9 I = 0.416 α = 1.452; N7 I = 0.411 α = 1.528; N3 I = 0.269 α = 0.858; N8 I = 0.371 α = 1.162; N2 I = 0.417 α = 1.590; N6 I = 0.309 α = 0.962) in PhyML 3.0 [11]. Statistical support for the phylogenies was assessed by the approximate likelihood test using a Shimodaira-Hasegawa-like procedure in PhyML 3.0. The topology of trees was visualized in FigTree 1.4. Viruses from different hosts are indicated by: wild birds, green; poultry, orange; human, pink. (ZIP) [file pone.0091887.s001.zip › Figure S1(d) N3.tif]

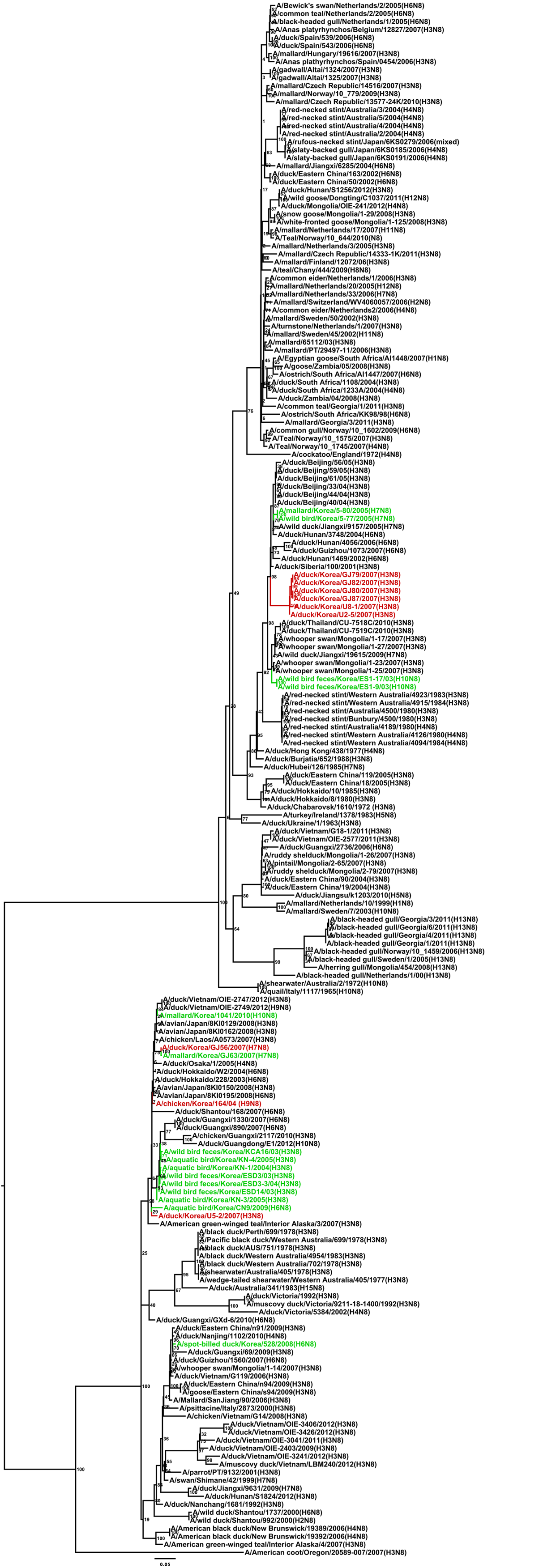

Supplement: Figure S1 — Phylogenies of seven surface genes: H7 ( n = 541) (a), N9 ( n = 179) (b), N7 ( n = 148) (c), N3 ( n = 273) (d), N8 ( n = 194) (e), N2 ( n = 191) (f), and N6 ( n = 381) (g). Tip and branch colors represent host origin (wild birds in green, domestic birds in red) of all of the Korean H7 viruses. Phylogenetic trees were constructed using the maximum likelihood method with general time-reversible model with invariant sites and 4 gamma-distributed heterogeneous substitution rates (GTR+ I + Γ4 model) and 100 bootstrap replications (H7 I = 0.285 α = 1.092; N9 I = 0.416 α = 1.452; N7 I = 0.411 α = 1.528; N3 I = 0.269 α = 0.858; N8 I = 0.371 α = 1.162; N2 I = 0.417 α = 1.590; N6 I = 0.309 α = 0.962) in PhyML 3.0 [11]. Statistical support for the phylogenies was assessed by the approximate likelihood test using a Shimodaira-Hasegawa-like procedure in PhyML 3.0. The topology of trees was visualized in FigTree 1.4. Viruses from different hosts are indicated by: wild birds, green; poultry, orange; human, pink. (ZIP) [file pone.0091887.s001.zip › Figure S1(e) N8.tif]

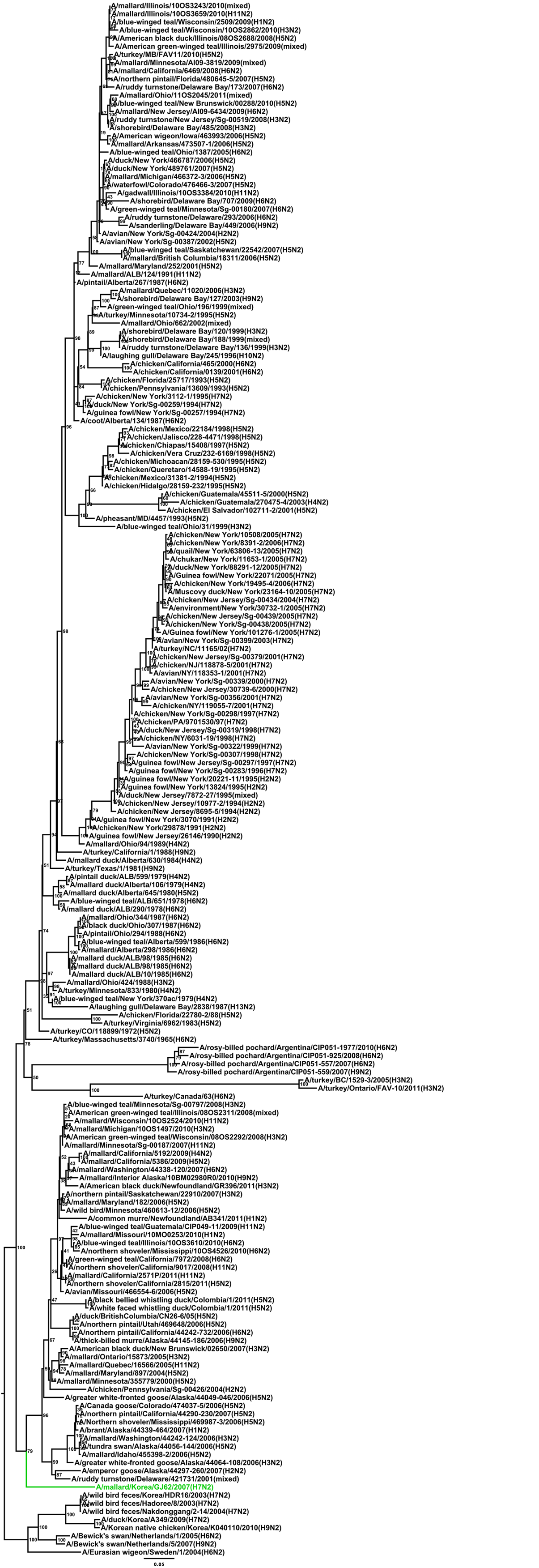

Supplement: Figure S1 — Phylogenies of seven surface genes: H7 ( n = 541) (a), N9 ( n = 179) (b), N7 ( n = 148) (c), N3 ( n = 273) (d), N8 ( n = 194) (e), N2 ( n = 191) (f), and N6 ( n = 381) (g). Tip and branch colors represent host origin (wild birds in green, domestic birds in red) of all of the Korean H7 viruses. Phylogenetic trees were constructed using the maximum likelihood method with general time-reversible model with invariant sites and 4 gamma-distributed heterogeneous substitution rates (GTR+ I + Γ4 model) and 100 bootstrap replications (H7 I = 0.285 α = 1.092; N9 I = 0.416 α = 1.452; N7 I = 0.411 α = 1.528; N3 I = 0.269 α = 0.858; N8 I = 0.371 α = 1.162; N2 I = 0.417 α = 1.590; N6 I = 0.309 α = 0.962) in PhyML 3.0 [11]. Statistical support for the phylogenies was assessed by the approximate likelihood test using a Shimodaira-Hasegawa-like procedure in PhyML 3.0. The topology of trees was visualized in FigTree 1.4. Viruses from different hosts are indicated by: wild birds, green; poultry, orange; human, pink. (ZIP) [file pone.0091887.s001.zip › Figure S1(f) N2.tif]

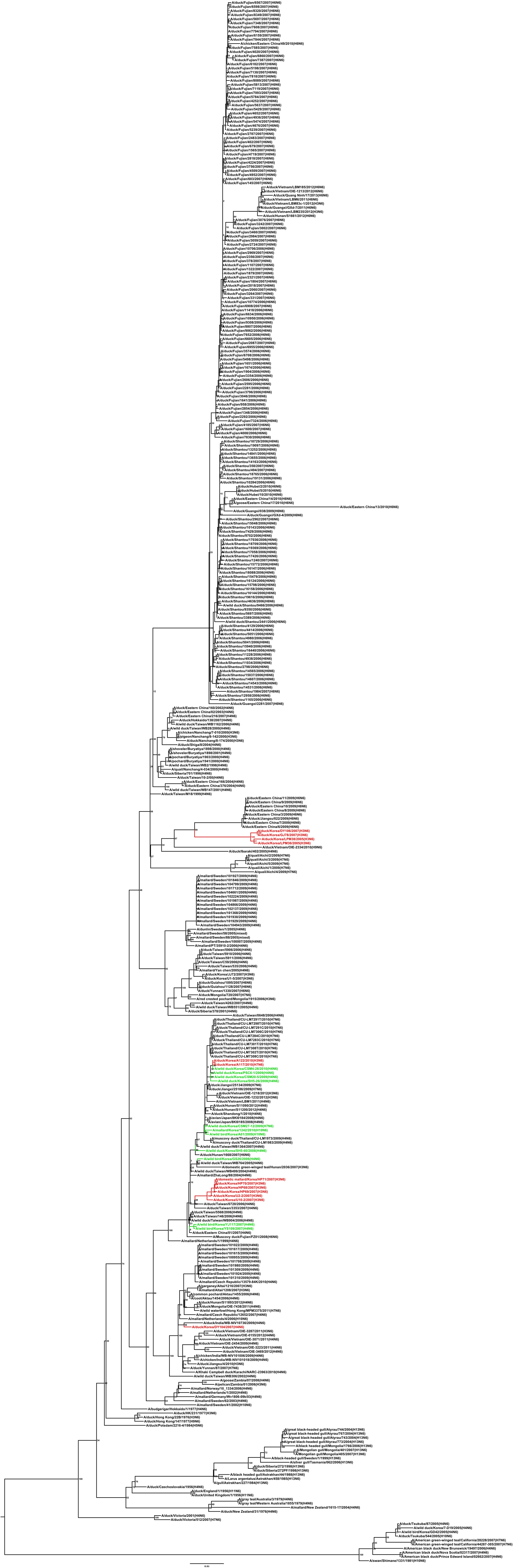

Supplement: Figure S1 — Phylogenies of seven surface genes: H7 ( n = 541) (a), N9 ( n = 179) (b), N7 ( n = 148) (c), N3 ( n = 273) (d), N8 ( n = 194) (e), N2 ( n = 191) (f), and N6 ( n = 381) (g). Tip and branch colors represent host origin (wild birds in green, domestic birds in red) of all of the Korean H7 viruses. Phylogenetic trees were constructed using the maximum likelihood method with general time-reversible model with invariant sites and 4 gamma-distributed heterogeneous substitution rates (GTR+ I + Γ4 model) and 100 bootstrap replications (H7 I = 0.285 α = 1.092; N9 I = 0.416 α = 1.452; N7 I = 0.411 α = 1.528; N3 I = 0.269 α = 0.858; N8 I = 0.371 α = 1.162; N2 I = 0.417 α = 1.590; N6 I = 0.309 α = 0.962) in PhyML 3.0 [11]. Statistical support for the phylogenies was assessed by the approximate likelihood test using a Shimodaira-Hasegawa-like procedure in PhyML 3.0. The topology of trees was visualized in FigTree 1.4. Viruses from different hosts are indicated by: wild birds, green; poultry, orange; human, pink. (ZIP) [file pone.0091887.s001.zip › Figure S1(g) N6.tif]

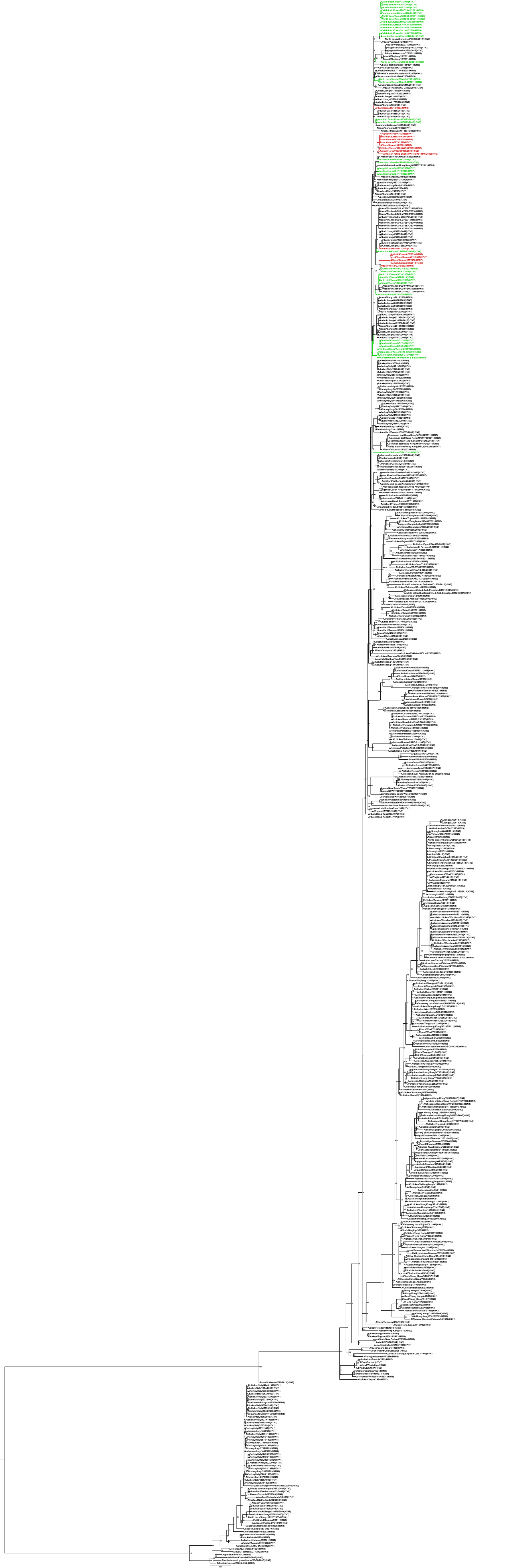

Supplement: Figure S2 — Phylogenies of six internal genes: PB2 ( n = 495) (a), PB1 ( n = 509) (b), PA ( n = 520) (c), NP ( n = 472) (d), M ( n = 520) (e), and NS ( n = 545) (f). Tip and branch colors represent host origin (wild birds in green, domestic birds in red) of all of the Korean H7 viruses. Phylogenetic trees were constructed using the maximum likelihood method with a general time-reversible model with invariant sites and 4 gamma-distributed heterogeneous substitution rates (GTR+ I + Γ4 model) and 100 bootstrap replications (PB2 I = 0.308 α = 0.749; PB1 I = 0.377 α = 0.899; PA I = 0.320 α = 0.773; NP 0.409 α = 0.874; M I = 0.146 α = 0.435; NS I = 0.161 α = 0.768) in PhyML 3.0 (Guindon et al., 2010). Statistical support for the phylogenies was assessed by the approximate likelihood test using a Shimodaira-Hasegawa-like procedure in PhyML 3.0. The topology of trees was visualized in FigTree 1.4. (ZIP) [file pone.0091887.s002.zip › Figure S2(f) NS.tif]

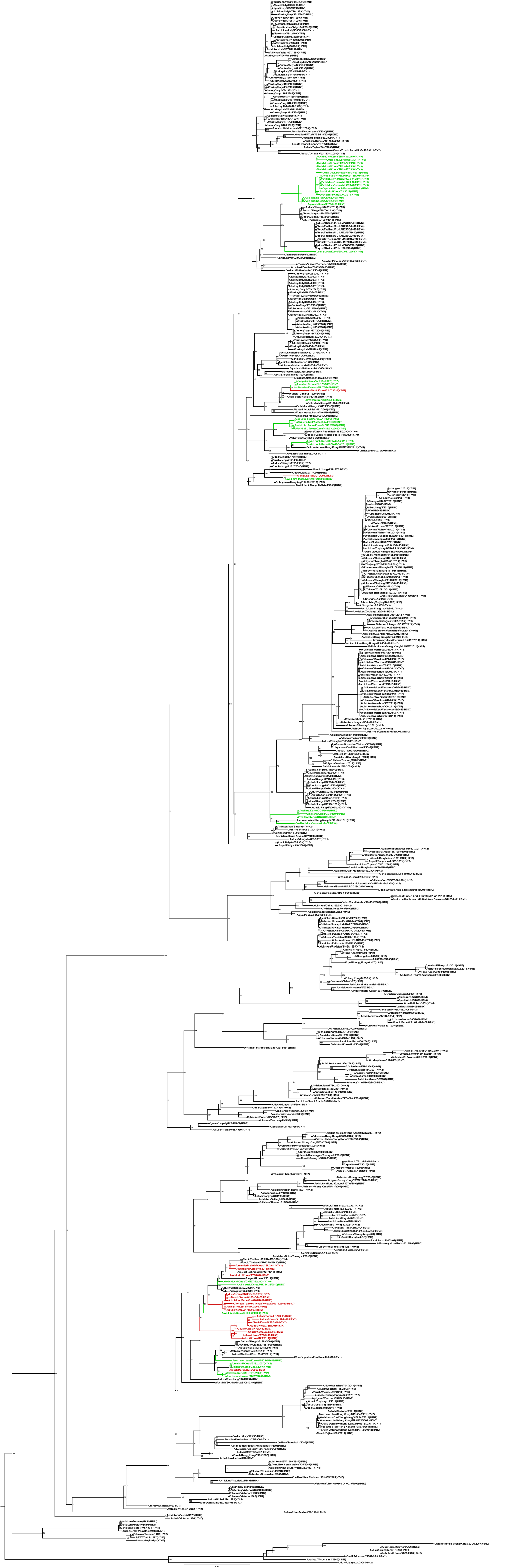

Supplement: Figure S2 — Phylogenies of six internal genes: PB2 ( n = 495) (a), PB1 ( n = 509) (b), PA ( n = 520) (c), NP ( n = 472) (d), M ( n = 520) (e), and NS ( n = 545) (f). Tip and branch colors represent host origin (wild birds in green, domestic birds in red) of all of the Korean H7 viruses. Phylogenetic trees were constructed using the maximum likelihood method with a general time-reversible model with invariant sites and 4 gamma-distributed heterogeneous substitution rates (GTR+ I + Γ4 model) and 100 bootstrap replications (PB2 I = 0.308 α = 0.749; PB1 I = 0.377 α = 0.899; PA I = 0.320 α = 0.773; NP 0.409 α = 0.874; M I = 0.146 α = 0.435; NS I = 0.161 α = 0.768) in PhyML 3.0 (Guindon et al., 2010). Statistical support for the phylogenies was assessed by the approximate likelihood test using a Shimodaira-Hasegawa-like procedure in PhyML 3.0. The topology of trees was visualized in FigTree 1.4. (ZIP) [file pone.0091887.s002.zip › Figure S2 (a) PB2.tif]

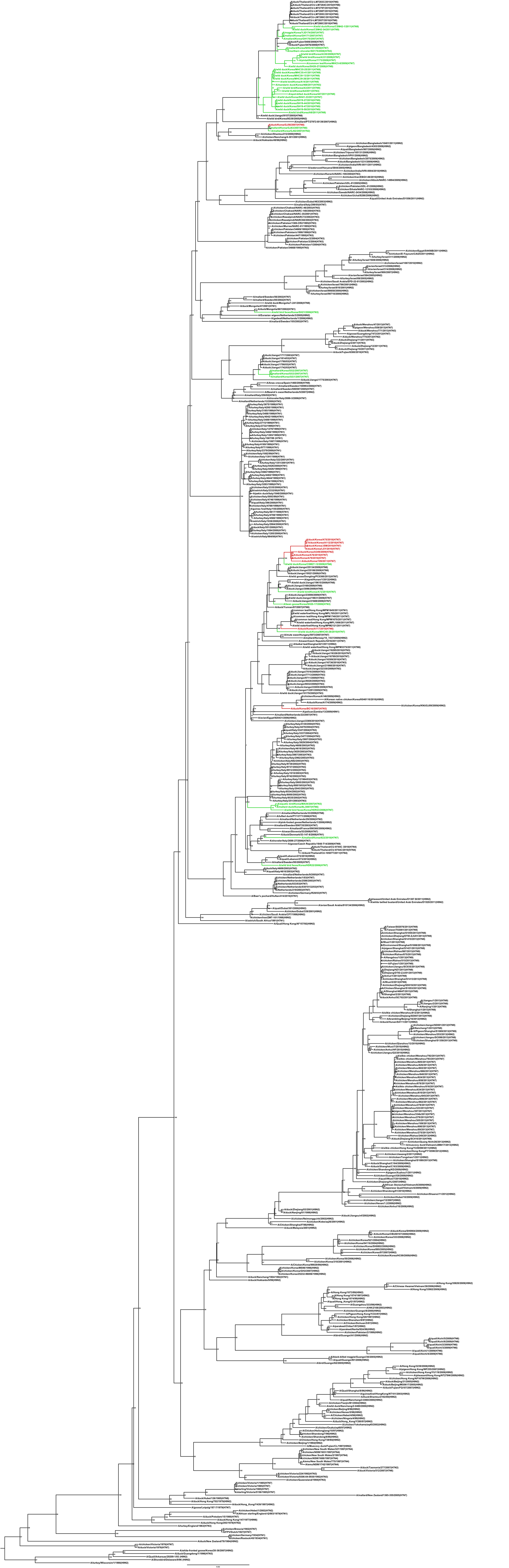

Supplement: Figure S2 — Phylogenies of six internal genes: PB2 ( n = 495) (a), PB1 ( n = 509) (b), PA ( n = 520) (c), NP ( n = 472) (d), M ( n = 520) (e), and NS ( n = 545) (f). Tip and branch colors represent host origin (wild birds in green, domestic birds in red) of all of the Korean H7 viruses. Phylogenetic trees were constructed using the maximum likelihood method with a general time-reversible model with invariant sites and 4 gamma-distributed heterogeneous substitution rates (GTR+ I + Γ4 model) and 100 bootstrap replications (PB2 I = 0.308 α = 0.749; PB1 I = 0.377 α = 0.899; PA I = 0.320 α = 0.773; NP 0.409 α = 0.874; M I = 0.146 α = 0.435; NS I = 0.161 α = 0.768) in PhyML 3.0 (Guindon et al., 2010). Statistical support for the phylogenies was assessed by the approximate likelihood test using a Shimodaira-Hasegawa-like procedure in PhyML 3.0. The topology of trees was visualized in FigTree 1.4. (ZIP) [file pone.0091887.s002.zip › Figure S2(b) PB1.tif]

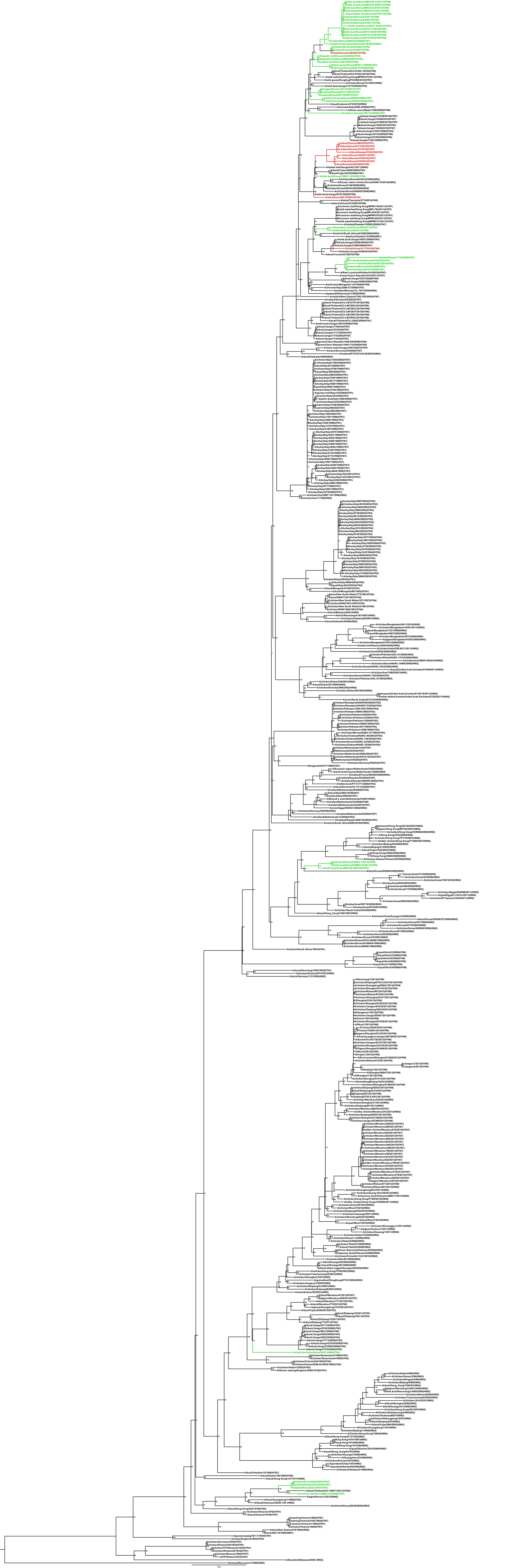

Supplement: Figure S2 — Phylogenies of six internal genes: PB2 ( n = 495) (a), PB1 ( n = 509) (b), PA ( n = 520) (c), NP ( n = 472) (d), M ( n = 520) (e), and NS ( n = 545) (f). Tip and branch colors represent host origin (wild birds in green, domestic birds in red) of all of the Korean H7 viruses. Phylogenetic trees were constructed using the maximum likelihood method with a general time-reversible model with invariant sites and 4 gamma-distributed heterogeneous substitution rates (GTR+ I + Γ4 model) and 100 bootstrap replications (PB2 I = 0.308 α = 0.749; PB1 I = 0.377 α = 0.899; PA I = 0.320 α = 0.773; NP 0.409 α = 0.874; M I = 0.146 α = 0.435; NS I = 0.161 α = 0.768) in PhyML 3.0 (Guindon et al., 2010). Statistical support for the phylogenies was assessed by the approximate likelihood test using a Shimodaira-Hasegawa-like procedure in PhyML 3.0. The topology of trees was visualized in FigTree 1.4. (ZIP) [file pone.0091887.s002.zip › Figure S2(c) PA.tif]

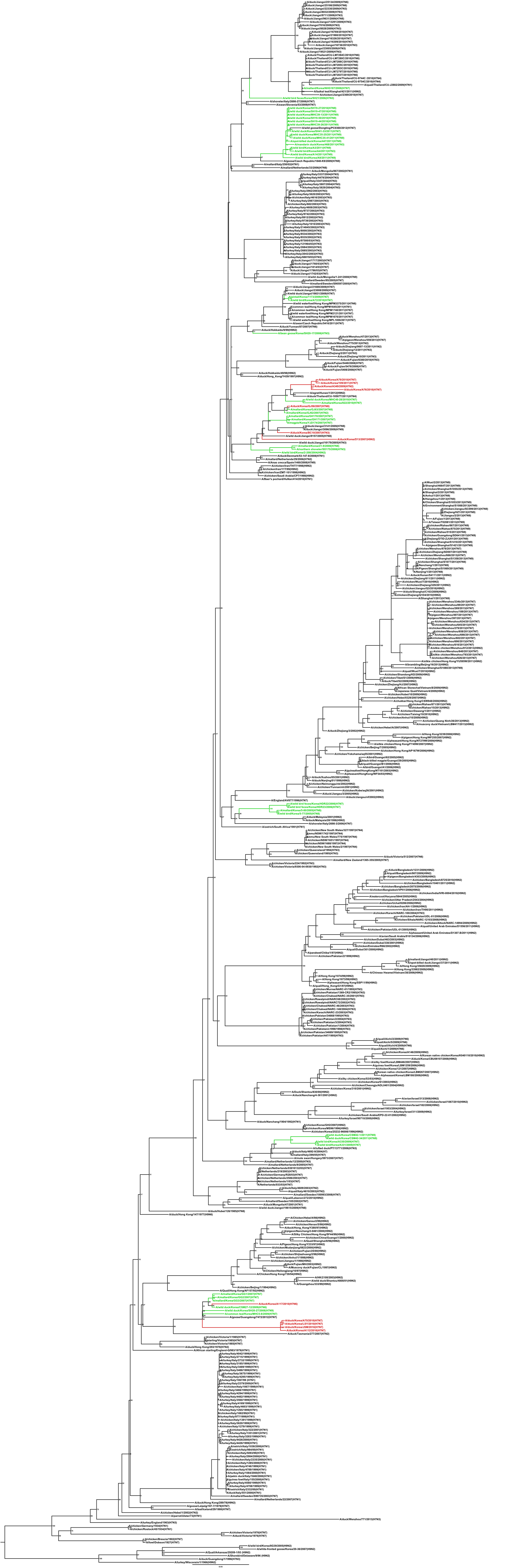

Supplement: Figure S2 — Phylogenies of six internal genes: PB2 ( n = 495) (a), PB1 ( n = 509) (b), PA ( n = 520) (c), NP ( n = 472) (d), M ( n = 520) (e), and NS ( n = 545) (f). Tip and branch colors represent host origin (wild birds in green, domestic birds in red) of all of the Korean H7 viruses. Phylogenetic trees were constructed using the maximum likelihood method with a general time-reversible model with invariant sites and 4 gamma-distributed heterogeneous substitution rates (GTR+ I + Γ4 model) and 100 bootstrap replications (PB2 I = 0.308 α = 0.749; PB1 I = 0.377 α = 0.899; PA I = 0.320 α = 0.773; NP 0.409 α = 0.874; M I = 0.146 α = 0.435; NS I = 0.161 α = 0.768) in PhyML 3.0 (Guindon et al., 2010). Statistical support for the phylogenies was assessed by the approximate likelihood test using a Shimodaira-Hasegawa-like procedure in PhyML 3.0. The topology of trees was visualized in FigTree 1.4. (ZIP) [file pone.0091887.s002.zip › Figure S2(d) NP.tif]

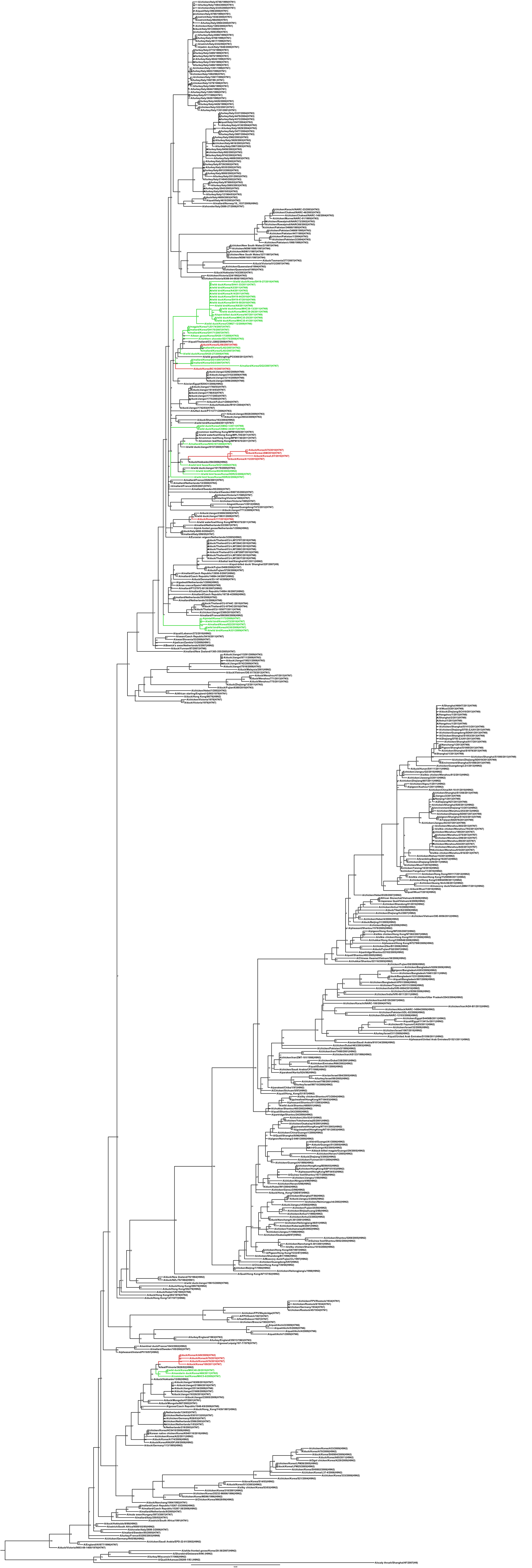

Supplement: Figure S2 — Phylogenies of six internal genes: PB2 ( n = 495) (a), PB1 ( n = 509) (b), PA ( n = 520) (c), NP ( n = 472) (d), M ( n = 520) (e), and NS ( n = 545) (f). Tip and branch colors represent host origin (wild birds in green, domestic birds in red) of all of the Korean H7 viruses. Phylogenetic trees were constructed using the maximum likelihood method with a general time-reversible model with invariant sites and 4 gamma-distributed heterogeneous substitution rates (GTR+ I + Γ4 model) and 100 bootstrap replications (PB2 I = 0.308 α = 0.749; PB1 I = 0.377 α = 0.899; PA I = 0.320 α = 0.773; NP 0.409 α = 0.874; M I = 0.146 α = 0.435; NS I = 0.161 α = 0.768) in PhyML 3.0 (Guindon et al., 2010). Statistical support for the phylogenies was assessed by the approximate likelihood test using a Shimodaira-Hasegawa-like procedure in PhyML 3.0. The topology of trees was visualized in FigTree 1.4. (ZIP) [file pone.0091887.s002.zip › Figure S2(e) M.tif]
